# Exploring the Role of E6 and E7 Oncoproteins in Cervical Oncogenesis through MBD2/3-NuRD Complex Chromatin Remodeling

**DOI:** 10.3390/genes15050560

**Published:** 2024-04-27

**Authors:** Alina Fudulu, Carmen Cristina Diaconu, Iulia Virginia Iancu, Adriana Plesa, Adrian Albulescu, Marinela Bostan, Demetra Gabriela Socolov, Irina Liviana Stoian, Raluca Balan, Gabriela Anton, Anca Botezatu

**Affiliations:** 1 Stefan S. Nicolau Institute of Virology, Romanian Academy, 030304 Bucharest, Romania; alina.fudulu@virology.ro (A.F.); iulia.iancu@virology.ro (I.V.I.); adriana.plesa@virology.ro (A.P.); adrian.albulescu@virology.ro (A.A.); marinela.bostan@virology.ro (M.B.); gabi_anton2000@yahoo.com (G.A.); anca.botezatu@virology.ro (A.B.); 2 Pharmacology Department, National Institute for Chemical Pharmaceutical Research and Development, 031299 Bucharest, Romania; 3 Department of Obstetrics and Gynecology, Grigore T. Popa University of Medicine and Pharmacy, 700115 Iasi, Romania; demetrasocolov@gmail.com (D.G.S.); stoian.irinalv@yahoo.com (I.L.S.); raluca12usa@yahoo.com (R.B.)

**Keywords:** NuRD complex, E6 and E7 oncoproteins, HPV, chromatin remodeling, ChIP-seq

## Abstract

Background: Cervical cancer is among the highest-ranking types of cancer worldwide, with human papillomavirus (HPV) as the agent driving the malignant process. One aspect of the infection’s evolution is given by epigenetic modifications, mainly DNA methylation and chromatin alteration. These processes are guided by several chromatin remodeling complexes, including NuRD. The purpose of this study was to evaluate the genome-wide binding patterns of the NuRD complex components (MBD2 and MBD3) in the presence of active HPV16 E6 and E7 oncogenes and to determine the potential of identified genes through an experimental model to differentiate between cervical precursor lesions, with the aim of establishing their utility as biomarkers. Methods: The experimental model was built using the CaSki cell line and shRNA for E6 and E7 HPV16 silencing, ChIP-seq, qRT-PCR, and Western blot analyses. Selected genes’ expression was also assessed in patients. Results: Several genes have been identified to exhibit altered transcriptional activity due to the influence of HPV16 E6/E7 viral oncogenes acting through the MBD2/MBD3 NuRD complex, linking them to viral infection and cervical oncogenesis. Conclusions: The impacted genes primarily play roles in governing gene transcription, mRNA processing, and regulation of translation. Understanding these mechanisms offers valuable insights into the process of HPV-induced oncogenesis.

## 1. Introduction

Cervical cancer is the fourth most common female cancer after breast, colorectal, and lung cancer. According to GLOBOCAN, in 2022, 662,301 new cases and 348,874 deaths were registered, most of them occurring predominantly in low- and middle-income countries [1]. Human papillomavirus (HPV) is considered the etiological agent of cervical malignancy. An important role in cervical oncogenesis is played by the two viral oncogenes (E6 and E7) of high-risk human papillomaviruses (hrHPV) genotypes. The proteins encoded by these genes interact with numerous cellular targets and induce oncogenesis mainly by degradation and inactivation of p53 and pRB tumor suppressors [2]. The main cause in cervical carcinogenesis is persistent infection with hrHPV types, with HPV16 and 18 being responsible for approximately 70% of invasive cervical cancers [3]. The progression from precursor lesions to invasive cervical cancer is caused by persistent hrHPV infections that induce changes in both host genome and epigenome that affect the transcriptional program [4]. Studies following the amplification of the host genome at the site of HPV integration and dysregulation of tumor suppressor genes by the HPV oncoproteins cannot fully explain all the gene expression changes in HPV cancers. This has led to the hypothesis that epigenetic modifications are an important mechanism in HPV-associated carcinogenesis [5]. These changes at epigenetic level involve DNA methylation, histone modifications, and non-coding RNAs (ncRNAs) activity, with the key players being represented by DNA methylation and histone acetylation. DNA methylation and histone modifications are closely interconnected, defining the chromatin state and collectively regulating gene expression [6]. These processes are controlled by several chromatin remodeling complexes (SWI/SNF, ISWI, NuRD/Mi-2/CHD, INO80, and SWR1). The nucleosome remodeling and deacetylation complex (NuRD) is a group of associated proteins with ATP-dependent chromatin remodeling and histone deacetylase activities [7]. This complex covers a multi-functional and highly conserved combination of chromatin-modifying activities comprised of nucleosome remodeling and histone deacetylation and contains seven subunits: HDAC1 and HDAC2 (histone deacetylase core proteins), the histone-binding proteins RbAp46 and RbAp48, the metastasis-associated proteins MTA1 (or MTA2/MTA3), methyl-CpG-binding domain protein MBD3 (or MBD2), and chromodomain-helicase-DNA-binding protein CHD3 (aka Mi-2alpha) or CHD4 (aka Mi-2beta). MBD2 and MBD3 recruit the NuRD complex in a mutually exclusive manner with different functions: while MBD2 binds to methylated DNA (at 5-methylcytosine—5mC), MBD3 binds to DNA at 5-hydroxymethylcytosine (5 hmC) [8]. It has been shown that HDAC2 interacts with E7 viral protein through Mi2β protein and mediates cell transformation [9].

This study aims to evaluate the genome-wide binding patterns of the NuRD complex components (MBD2, MBD3) in the presence of HPV16 E6/E7 oncogene activity using ChIP-seq techniques. Additionally, we seek to determine the potential of genes identified through this experimental model to differentiate between cervical precursor lesions, with the aim of establishing their utility as biomarkers. Also, the localization of the MBD2/MBD3 NuRD Complex in the context of viral infection will provide new perspective on comprehending the cellular and molecular pathways influenced by HPV16 E6/E7 oncoproteins. Given the NuRD complex’s propensity to impact tumorigenesis by altering the expression and function of transcription factors, our investigation revealed valuable insights into this process [10].

## 2. Materials and Methods

### 2.1. Cell Culture and Culture Conditions

The CaSki cell line was selected and purchased from the American Type Culture Collection (ATCC) and cultured according to the ATCC recommendations. CaSki, an HPV-positive cervical carcinoma cell line, is reported to contain an integrated human papillomavirus type 16 genome (HPV16, about 600 copies per cell) as well as sequences related to HPV18. The cell line was maintained in RPMI1640 medium (Gibco/Life Technologies, Waltham, MA, USA) supplemented with 10% fetal bovine serum and grown at 37 °C with 5% CO_2_.

### 2.2. Patient Sample Collection

To assess the expression levels of specific genes, we included in our study 74 cervical specimens from women who self-referred for gynecological examinations in “Cuza Voda” Clinical Hospital of Obstetrics and Gynecology from Iasi. This study was conducted in accordance with the principles outlined in the Declaration of Helsinki. Written informed consent was obtained from all participants prior to their involvement.

### 2.3. HPV DNA Detection and Genotyping

DNA extraction from cervical samples was carried out using the QIAamp DNA Mini Kit (Qiagen, Hilden, Germany) following the manufacturer’s guidelines. The concentration and purity of each DNA sample were assessed using a NanoDrop ND-1000 spectrophotometer (Thermo Fisher Scientific Inc., Waltham, MA, USA). Detection and genotyping of human papillomavirus (HPV) were performed using the INNO-LiPA^®^ HPV Genotyping Extra II kit (Fujirebio Europe, Ghent, Belgium), following the manufacturer’s protocol. This kit includes 32 HPV genotype-specific probes, the SPF10 primer set, human DNA control primers, and pre-prepared amplification reagents for HPV genotyping. The method facilitates the categorization of samples into high-risk (hrHPV), low-risk (lrHPV), and undetermined risk HPV types. The test covers 13 high-risk types: 16, 18, 31, 33, 35, 39, 45, 51, 52, 56, 58, 59, and 68.

### 2.4. Design for E6/E7 HPV16 shRNAs, Plasmid Construction and Cloning

The development of plasmids that express shRNA specifically targeting E6 and E7 involved a series of steps. Initially, shRNA oligonucleotides are designed to specifically target particular regions within the E6 and E7 gene transcripts. Subsequently, these oligonucleotides were incorporated into a plasmid vector designed for shRNA expression. Using Clontech software (In-Fusion Cloning Primer Design Tool v1.0, Takara, San Jose, CA, USA), a specialized program with specific algorithms to build shRNA sequences presenting high efficiency and also facilitating their introduction into transfection (vector), specific shRNA sequences for the targeted genes (E7 or/and E6 HPV16) were designed and were subsequently synthesized by LifeTechnology (Waltham, MA, USA). To facilitate the transfection of CaSki cells, the custom-designed shRNA sequences were cloned using the BLOCK-iT U6 RNAi Entry Vector kit (LifeTechnology, Waltham, MA, USA). The kit allows a fast and efficient cloning of specific shRNA molecules using the Gateway technology (Invitrogen, Waltham, MA, USA), which is based on the bacteriophage λ property of site-specific recombination to introduce a DNA sequence of interest in a vector by enzymatic methods.

### 2.5. Plasmid DNA Isolation and Sanger Sequencing

Plasmid DNA isolation and purification were performed using HiSpeed Plasmid Purification Maxi Kit (Qiagen, Hilden, Germany) according to the manufacturer’s protocol. To confirm shRNA sequences, the constructs were sequenced for the selected regions using U6 Forward 5′-GGACTATCATATGCTTACCG-3 and M13 Reverse 5′-CAGGAAACAGCTATGAC-3′ primers from the kit. The region of interest was amplified using classic PCR. Further, amplicons were excised from the gel after electrophoresis in 1% agarose, and DNA was extracted using QIAquick Gel Extraction Kit (Qiagen, Hilden, Germany) according to the manufacturer’s instructions. The next step involved another round of amplification of the region of interest using the BigDye^TM^ (Life Technologies, Waltham, MA, USA) Terminator v3.1 Cycle Sequencing Reagent (Applied Biosystems^TM^, Waltham, MA, USA). Before running the sequencing program, the amplicons obtained were further purified using BigDye XTerminator^TM^ Purification Kit (Applied Biosystems^TM^, Waltham, MA, USA) according to the manufacturer’s instructions. The sequencing was performed on ABI PRISM 3130 Genetic Analyzer and the results were evaluated with BioEdit v7.2 and NCBI Blast software (https://blast.ncbi.nlm.nih.gov/Blast.cgi, accessed on 24 April 2024).

### 2.6. Transfection of E6 and E7 shRNAs in CaSki Cells (Experimental Model)

In total, 1.5 × 10^5^ CaSki cells were cultured in 25 cm^3^ flasks in RPMI 1640 medium (Gibco) supplemented with 10% fetal serum and 1% L-Glutamine, without antibiotic, until the cells reached 70% confluence. CaSki cells were transfected with three different concentrations (4, 6, and 8 µg) of the five constructs in presence of Lipofectamine 2000 (Invitrogen) and OptiMEM (Invitrogen), following manufacturer’s recommendations. In the same experiment, untreated cells were used as cell controls, and transfection medium-treated cells were used as controls as well. Four hours later, lipofectamine was replaced with fresh complete RPMI medium. Transfection efficiency estimation was performed comparing the five constructs by assessing E6 and E7 mRNA expression levels in transfected cell cultures vs. controls at 24 and 48 h. Total RNA was isolated from cells using QIAamp Viral RNA Mini Kit (Qiagen) and qualitatively and quantitatively assessed for each sample. For cDNA synthesis, High-Capacity cDNA Reverse Transcription Kit (Thermo Fisher Scientific Inc.) was used. E6 and E7 mRNAs were quantified in quantitative real-time PCR (qRT-PCR) using specific primers and Maxima SYBR Green/ROX qPCR Master Mix (2X) (Thermo Fisher Scientific). All samples were tested in triplicate. In order to determine the mRNA level of E6 and E7 HPV16 following treatment with specific shRNA, a relative quantification method was used (ΔCq) using GAPDH as the reference gene. The obtained values were further normalized also to controls (untreated cells CaSki) (ΔΔCq). The shRNA transfection efficiency was used with the formula: % knockdown = 100 − 100 × 2^−ΔΔCq E7/E6 HPV16^; ΔCq = Cq _E7/E6 HPV16_ − Cq _GAPDH_; ΔΔCq = ΔCq _E7/E6 HPV16_ − ΔCq _cells controls_

### 2.7. Evaluation of Cell Cycle, Proliferation, Apoptosis, and Senescence in Transfected Cells

After the inhibition of the E6 and/or E7 oncogene, CaSki tumor cells were trypsinized, washed in a cold PBS solution, and fixed with 70% ethanol for at least 24 h at 4 °C. Then, the cells were treated with 100 μg/mL RNase A for 10 min and stained with 50 μg/mL propidium iodide (PI) another 15 min. The samples were stored at 4 °C until analyzed by flow cytometry. A minimum of 20,000 events for each sample were acquired using a FACS CantoII flow cytometer. The cell cycle distribution was analyzed using ModFIT software v3.2 (BD Biosciences, San Jose, CA, USA) after debris exclusion [11]. For apoptosis analysis, CaSki tumor cells treated to inhibit E6 and/or E7 oncogene, detached using trypsin-EDTA (0.25%) solution, and centrifuged 10 min at 200× *g* were resuspended in cold binding buffer.

The apoptosis assay was carried out with the Annexin V-FITC Apoptosis Detection Kit (BD Biosciences, San Jose, CA, USA) according to the manufacturer’s protocol. A total of 1 × 10^5^ cells/sample in 100 µL binding buffer were stained simultaneously with 5 µL FITC-Annexin V (green fluorescence) and 5 µL propidium iodide (PI) in the dark, at room temperature for 15 min. A total of 400 µL of Annexin V binding buffer was added and 10,000 cells/sample were acquired using a BD Canto II flow cytometer. The analysis was performed using DIVA 6.2 software to discriminate viable cells that are negative both for FITC and PI staining (FITC−PI−) from necrotic cells (FITC−PI+), late apoptotic cells that are double-stained for both fluorochromes (FITC+PI+), and early apoptotic cells (FITC+PI−) [12].

### 2.8. Chromatin Immunoprecipitation (ChIP) and Sequencing

For optimal ChIP results, we used approximately 4 × 10^7^ cells for each immunoprecipitation to be performed. Protein–DNA cross-linking ChIP-DNA was prepared using SimpleChIP Enzymatic Chromatin IP Kit #9003 (Cell Signaling Technology, Danvers, MA, USA) following manufacturer’s protocol. Chromatin was digested and sonicated (Bioruptor^®^ Pico-Diagenode, Liege, Belgium) into 150–900 bp DNA/protein fragments. To achieve optimal ChIP results, 5 to 10 μg of digested, cross-linked DNA are used for immunoprecipitation. Antibodies used were anti-MBD2+MBD3 antibody [106B691]—ChIP Grade (ab45027). For positive control, 10 μL of Histone H3 (D2B12) XP Rabbit mAb # 4620 was used, and 2 μL of Normal Rabbit IgG # 2729 was added for negative control. Samples were incubated overnight at 4 °C with orbital rotation. As a final step, the protein–DNA cross links were reversed and the DNA was purified using spin columns. DNA samples enriched by ChIP will be used in the next phase for sequencing library preparation on the NGS platform MiSeq-Illumina. For this, a TruSeq ChIP Library Preparation Kit from Illumina (San Diego, CA, USA) was used. The protocol was optimized for 5–10 ng input ChIP DNA. The manufacturer recommends normalizing the ChIP DNA samples to a final volume of 50 μL at 100–200 pg/μL. Indexed DNA libraries were normalized to 10 nM, and then pooled in equal volumes. DNA libraries not intended for pooling were normalized to 10 nM without pooling.

### 2.9. Quantitative Real-Time PCR

Total RNA extraction from patients’ samples was performed using TRIzol™ Reagent (Thermo Fisher Scientific, Waltham, MA, USA) and purified with RNeasy Mini kit (Qiagen, Hilden, Germany) according to the manufacturer’s instructions. The obtained RNA was reverse-transcribed into cDNA using High-Capacity cDNA Reverse Transcription Kit (Thermo Fisher Scientific, Waltham, MA, USA) according to the manufacturer instructions and stored at −20 °C until further use. RT-PCR was performed using Maxima SYBR Green/ROX qPCR Master Mix (2X) (Thermo Fisher Scientific, Waltham, MA, USA) on CFX96™ Real-Time PCR Detection System (BioRad, Hercules, CA, USA). The expression level of each investigated gene was normalized using GAPDH gene as reference, and each sample was analyzed in triplicate. Specific primers were designed for targeted genes using primer-BLAST (www.ncbi.nlm.nih.gov/tools/primer-blast/, accessed on 19 April 2023), and primer sequences are presented in Supplementary Table S2. The qPCR data were analyzed, and relative expression was calculated with Cq (quantification cycle) using 2^−∆Cq^/2^−ΔΔCq^ method.

### 2.10. Western Blot Analysis

Protein extraction for Western blot analysis was conducted on transfected and control cells using the M-PER™ reagent (Thermo Fisher Scientific, Waltham, MA, USA), following the manufacturer’s instructions. The protein concentration in the samples was determined using the Pierce™ Coomassie Bradford Protein Assay Kit (Thermo Fisher Scientific, Waltham, MA, USA). Approximately 35 µg of protein extract were then separated by 12.5% SDS-PAGE and transferred to an Immobilon-P PVDF membrane (Merck Millipore, Billerica, MA, USA). The primary antibodies used for Western blot were the following: Anti-β Actin antibody (ab8227) 1/1000 dilution; Anti-Human Papillomavirus 16 (E7) antibody [TVG 701Y] (ab20191) 1/250 dilution; Anti-HPV16 E6+HPV18 E6 antibody [C1P5] (ab70) 1/250 dilution. Secondary antibody conjugated to a reporter molecule was then used to identify the target proteins using chemiluminescent detection. For densitometry analysis, ImageJ software v1.54f (National Institute of Health, Bethesda, MD, USA) was used. All experiments were carried out in triplicate.

### 2.11. Bioinformatic Analysis and Data Visualization

ChIP-seq raw data (FASTQ files) were uploaded to Illumina BaseSpace ChIPSeq App, a pipeline who integrates MACS2 (Model-based Analysis of ChIP-Seq) and HOMER (Hypergeometric Optimization of Motif EnRichment) motif and annotation [13,14,15,16,17]. Data were trimmed and quality filtered using FastQC. The sequenced reads were mapped with Bowtie2, the output format for map files was BAM [18]. After alignment, reads mapped to the same genomic positions were filtered as redundant. The peak calling algorithm MACS2 was used to identify enriched regions representing protein–DNA interactions. HOMER was used for motif enrichment analysis by assessing the enrichment of known DNA sequence motifs within the peaks compared to a background set of genomic sequences and control samples. Annotated identified peaks (BAM files) with nearby genomic features were visualized using external genome browsers like IGV v2.16.0 (Integrative Genomics Viewer, San Diego, CA, USA) and EaSeq v1.2 (Copenhagen, Denmark). EaSeq facilitates the analysis of ChIP-seq data, allowing identification of the genomic regions bound by specific DNA-binding proteins. EaSeq was used for individual genomic loci visualization.

### 2.12. Pathway, Network and Functional Analysis

QIAGEN’s Ingenuity Pathway Analysis (IPA) was used for data mining and the generation of connectivity mapping between the most significant genes in patient samples, based on the manually curated publications stored in the QIAGEN Ingenuity Knowledge Base (QKB). Starting with molecules of interest, the Grow feature facilitates the discovery, addition, and connection of additional molecules relevant to a pathway. Thus, a list with selected genes was created in the IPA datasets. Forward, IPA’s “Build”–“Grow to Diseases and Functions” tool was used. This allows a rapid identification of diseases and functions that are significant to a set of Pathway molecules—in our case, the set of genes. Subsequently, a table was generated, showcasing a list of relevant diseases and functions associated with one or more pathway molecules. The list was ordered based on statistical significance determined using Fisher’s Exact Test (right-tailed). Moreover, the “Build”–“Grow to Canonical Pathways” tool was chosen, because this feature allows you to discover, include, and link other molecules of interest to a pathway. For this, every gene was selected to add all canonical pathways that consider only molecules and/or relationships where: “species = human” AND “disease = Cancer” OR Reproductive System Disease OR Infectious Disease. We also used the “Build”–“Path Explorer” tool, which helps identify the shortest path between nodes, being used to discover single or multi-step molecular paths between molecules or between molecules and diseases and functions of interest. Moreover, the tool enables the design of custom signaling pathways endorsed by the literature, starting with one or more genes, proteins, diseases, or biological functions of interest. At the end, we used again the “Build”–“Grow to Diseases and Functions” tool to identify the diseases and functions that are significant to the new set of molecules.

### 2.13. Statistical Analysis

The values were expressed as medians, and for statistical analyses: one-way ANOVA nonparametric (Kruskal–Wallis) test, nonparametric-test (Mann–Whitney), and paired *t*-test. GraphPad Prism version 9.3 software (Graph Pad Software Inc., San Diego, CA, USA) was used. *p* values < 0.05 were considered statistically significant.

**Figure 1:**
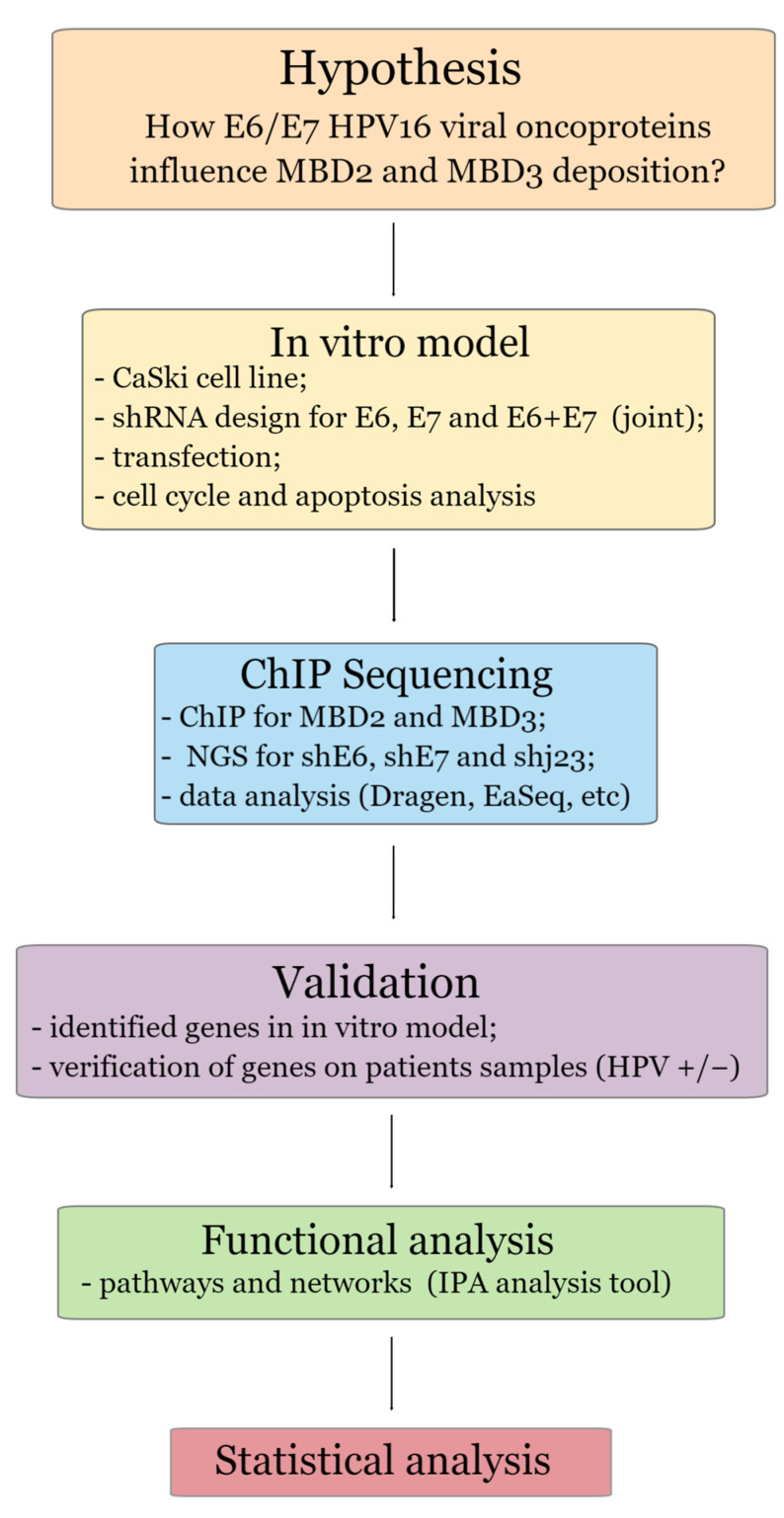
Flow diagram of the most important steps of the experimental work.

A detailed flow diagram outlining every stage from material and methods to the presentation of results was presented in Figure 1.

## 3. Results

### 3.1. Selection of shRNA Sequences for E6/E7 Viral Genes Silencing

In order to inhibit the expression of HPV16 E6 and E7 genes, we formulated five shRNA oligonucleotides directed at various sites within the HPV16 E6 or E7 transcript. (Table 1). The designed sequences target the HPV16 E7 coding region in exon 2 and the E6 coding region intron 1 of the HPV16E6E7 bicistronic transcripts, respectively. Also, we targeted both oncoproteins (E6^E7 joint) exon 2 near the ENH-1 splicing enhancer sequence [19]. The sequences are provided in Table 1.

Further, we cloned E6/E7 shRNA sequences into a pENTR™/U6 expression vector, along with scrambled shRNA 5′-CTTACAATCAGACTGGCGA-3′. For that, 200 μM of “Top strand” DNA oligo and 200 μM of “Bottom strand” DNA oligo of every construct were needed (Supplementary Table S1). The product obtained after the ligation reaction is further used for transforming competent *E. coli* cells, with pUC19 plasmid being used as a positive control. The shRNA cloned sequences were confirmed by Sanger sequencing and used in the further step.

### 3.2. E6 and E7 Viral Gene Silencing Using shRNA Sequences

**Figure 2:**
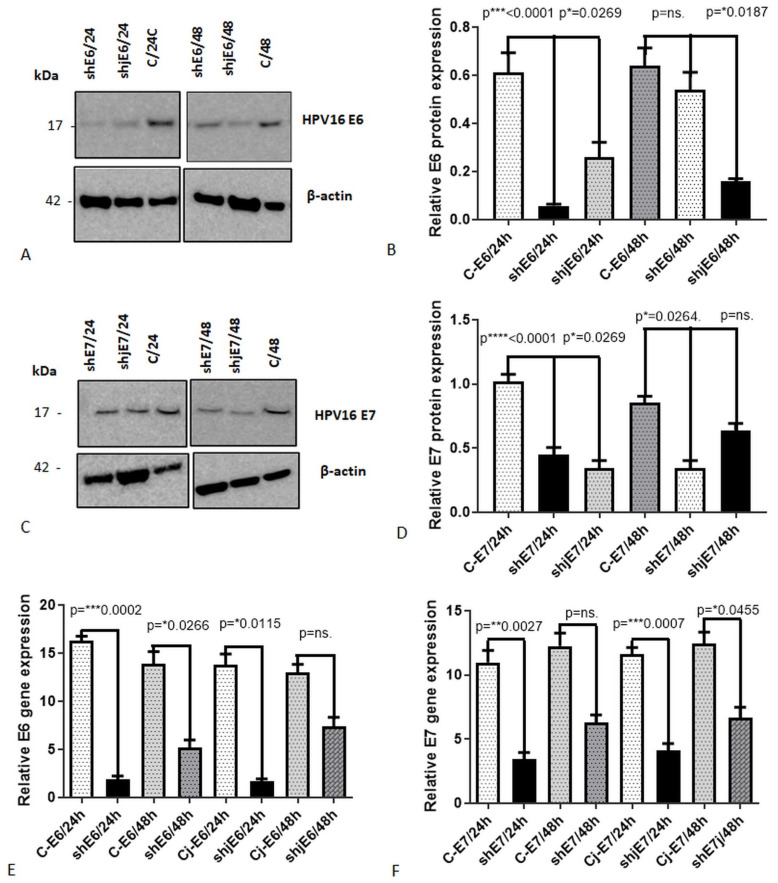
The impact of E6 and E7 shRNAs on the mRNA expression of HPV16 E6 and E7, assessed through RT-PCR, and their effect on protein expression analyzed via Western blot analysis (WB) after 24 and 48 h for treated and control (**C**) samples for: HPV16 E6 (**A**) and HPV16 E7 (**C**), densitometry analysis for E6HPV16 protein (**B**) and E7HPV16 protein (**D**), and relative gene expression by qRT-PCR of HPV16E6 (C, Cj—control joint) (**E**) and HPV16E7 (**F**). *—*p* < 0.05; **—*p* < 0.01; ***—*p* < 0.001; ****—*p* < 0.0001; ns—*p* > 0.05.

The shRNA constructs were used to transiently transfect CaSki cells with Lipofectamine. We assessed transfection efficiency by measuring the expression levels of E6/E7 mRNA in transfected cell cultures. Additionally, Western blot analysis was conducted to evaluate the expression of E6 and E7 proteins. To determine the optimal silencing protocol, we tested five different shRNA constructs at two time points (24 and 48 h) and three shRNA concentrations: C1 = 4 µg, C2 = 6 µg, and C3 = 8 µg. Upon evaluating the efficiency of the five specific shRNA constructs, we observed a variation in transfection efficiency in CaSki cells ranging from 30.26% to 80.45%. After all the experiments were carried out with shRNA mentioned in Supplementary Table S1, CaSki cells were transfected individually with shE6RNA_208, shE7RNA_158, and E6^E7 joint shjRNA 23 (shjRNA_23), being the most efficient at a concentration of 6 µg, 24 h post-transfection. Three shRNA (shE6rna_208; shjrna_23; shE7rna_158) were the most efficient at a concentration of 6 µg and 24 h post-transfection (Figure 2).

CaSki cells were transfected with shRNA E6(shE6rna_208), shRNA E7 (shE7rna_158), and E6^E7joint shjRNA 23 (shjrna_23).

### 3.3. Evaluation of Apoptosis and Cell Cycle Phases in CasKi Transfected Cells

The effect of E6 and E7 oncogenes knockdown was analyzed on the cell cycle in CasKi cells. The obtained results show that the blocking of the oncogene E6 or E7 individually in CaSki cells causes at 24 h a slight increase in the G1 phase (E6—62.6%/E7—62.6%) of the cell cycle accompanied by a decrease in the G2+M phase (E6—1.8%/E7—0.3%) and without significantly changing the S phase (E6—35.5%/E7—37.1%) compared to control cells (Control—G1: 55%; G2+M: 10.8%; S: 34.2%). After 48 h, the effect induced by blocking the E6 or E7 oncogene does not cause significant changes in the cell cycle phases compared to control cells (Control). When both oncogenes (shjRNA_23) were blocked in CasKi cells, the effect recorded at 48 h shows significant changes in the cell cycle, registering an increase in the G1 phase (69.4% versus 53.5% Control) and especially in the S phase (14.1% versus 46% Control), accompanied by a cell cycle block in the G2+M phase (17.5% versus 0.5% Control) (Supplementary Table S3 and Figure S1).

**Figure 3:**
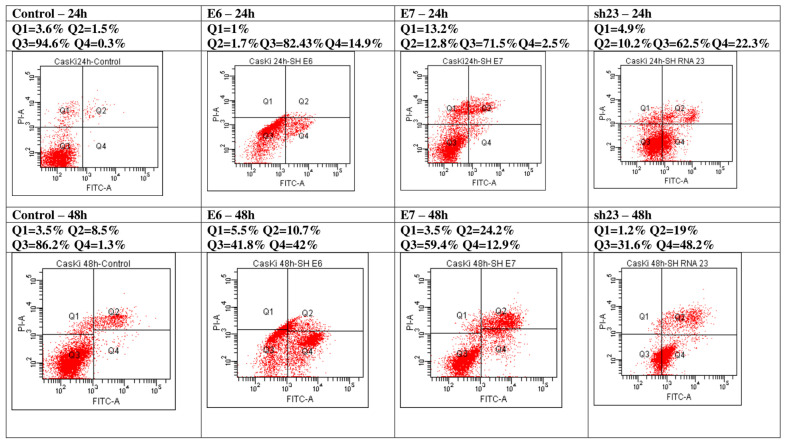
Flow cytometric analysis of cell viability and apoptosis processes in CaSki cells treated with shE6RNA, shE7RNA, shj23RNA, and untreated, after 24 and 48 h. The results are presented after the acquisition of data by flow cytometry (Q1 = necrosis, Q2 = late apoptosis, Q3 = viable cells, Q4 = early apoptosis).

Effects of the inhibition of E6 and/or E7 oncogenes on apoptosis of CaSki tumor cells were evaluated by flow cytometry assay using an Annexin V—FITC staining in a time-dependent manner. Recording the apoptosis of CaSki tumor cells after 24 h inhibition of one of the oncogenes E6 or E7 showed an increase of apoptosis from 1.8% in the control group, compared to 16.6% when the E6 is inhibited and 15.3% for the cells with E7 inhibited. The CaSki tumor cells treated to inhibit simultaneously both oncogenes E6 and E7 (shjRNA 23) entered apoptosis at a percentage of 32.5% (Figure 3).

These data show that the inhibition of both E6 and E7 oncogenes favors the entrance of cells in the apoptotic process in a higher percentage than when E6 or E7 oncogenes were inhibited separately. Favoring the entry of CaSki tumor cells into the apoptotic process as a result of the inhibition of the E6 and/or E7 oncogenes was also registered in the case of achieving the inhibition of the oncogenes after 48 h. G2+M phase of the cell cycle arrest of CaSki tumor cells suggests that DNA repair is incomplete and thus the cells with reduced repair capacity will enter programmed cell death. The results indicate that the inhibition of E6 and/or E7 oncogenes may contribute to the G2/M cell cycle arrest and the induction of apoptosis in CaSki tumor cells.

### 3.4. ChIP-Seq Analysis

To investigate the function of the NuRD complex on the genome level, we performed ChIP assays in CaSki cells for MBD2 and MBD3. To release soluble chromatin, the protocol uses micrococcal nuclease digestion, followed by sonication and immunoprecipitation (IP). FastQC tool was used to assess the data quality using FASTQ format as input. This format contains nucleotide sequences along with quality scores representing the confidence of each base call. The sequences were trimmed, and low-quality bases and adapter sequences were removed using tools like Trimmomatic v0.39. ChIP-seq libraries were filtered for adaptor sequences and aligned to the human reference genome UCSC assembly hg19, GRCh37 using Bowtie v1.3.1 Duplicate reads were removed with Picard tools v2.27.0. All the ChIP-Seq datasets passed the quality check with >84.73% of reads mapped to the reference genome for all replicates of CaSki untreated and >71.04 in shRNA treated used in the study. Using MACS2 v2.2.7.1 analysis, we identified significant peaks using a q-value cutoff of 0.01. The number of peaks in control samples varied from 12,355 to 27,081, while for different shRNA-treated samples, the number varied from 16,171 to 215,482.

### 3.5. Genome-Wide Characterization of MBD2, MBB3-NuRD Interactions

**Figure 4:**
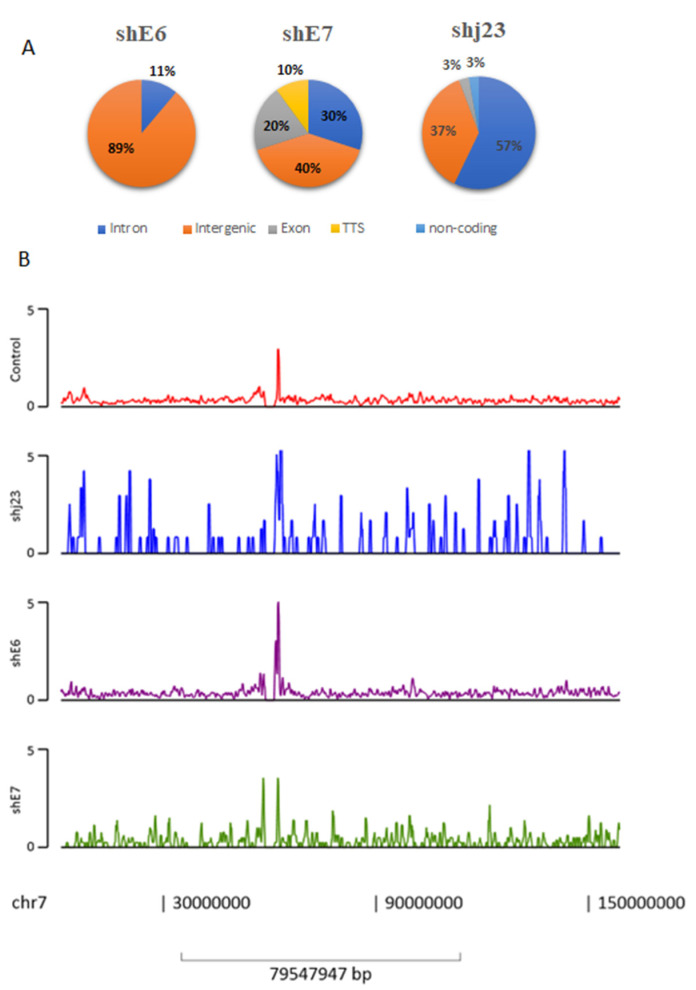
(**A**) MBD2/MBB3-NuRD Complex enriched region binding peaks. (**B**) Peaks distribution across the chromosome 7 in shRNAs treated vs. untreated CaSki cell line.

Enriched regions for MBD2 and MBD3 were called with default parameters using DNA input as control and retaining all statistically enriched regions (FDR < 1%). Peaks were annotated with nearby genes or genomic features using HOMER. MBD2, MBD3-NuRD peaks were numerous at intergenic enhancers, intronic sites, exon region, gene promoters, and TSS. As we observed in Figure 4, shRNA E6 treatment determines deposition of NuRD complex at the intergenic region (89%) in particular, while for shj23, NuRD deposition is in the intron region (57%). Interestingly, shRNA E7 treatment determines NuRD deposition to intergenic regions (40%) and also in intron (30%) and exon (20%) regions, along with TSS (10%). We also included, as a representative image, the peak distribution along chromosome 7, where one of the most important genes (miR-490) from our study was found (Figure 4).

### 3.6. Peak Annotation

Peak distribution varies between shRNA treatments as shRNA E6 and shjRNA 23 exhibit a higher number of different peaks as compared to control. Using the EaSeq genome browser, data were visualized regarding ChIP-seq read coverage along the genome. For quality check, ChIP-seq profiles must be valid and the data must be generated in concert with the modENCODE. We performed "Phantom peaks" analyses. "Phantom peaks" do not correspond to true protein–DNA interactions but rather result from technical biases or experimental noise. Understanding and addressing phantom peaks is crucial for accurate interpretation of ChIP-seq results. Our result showed a normal peak distribution with a good quality score (Supplementary Figure S2).

**Figure 5:**
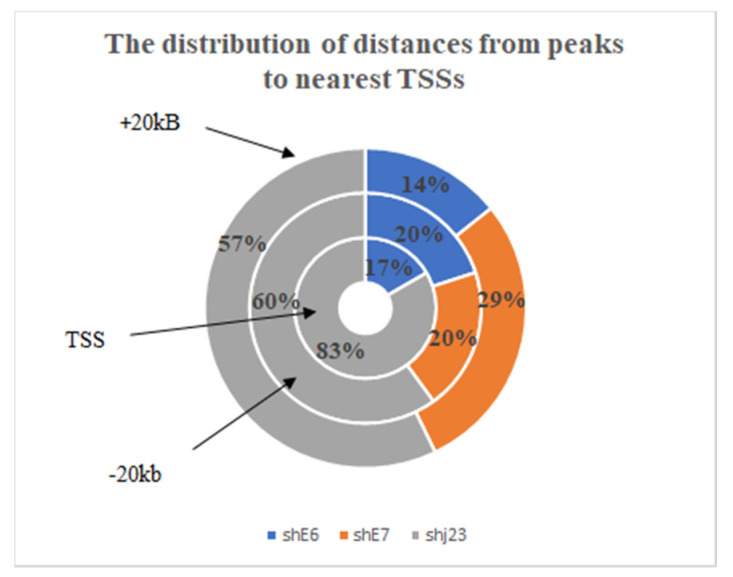
The distribution of distances from peaks to nearest TSSs in each of the treated samples; the circle closest to the center represents the distribution of peaks that are located in the TSS region, followed by two circles that represent the distribution of peaks located at +20 kB and −20 kB from the TSS region in treated samples.

Moreover, peak locations and intensity were also visualized. We identified regions of MBD2, MBD3 enriched binding, and we observed that ChIP-seq tags were obviously enriched in the intergenic regions (*n* = 25), introns (*n* = 24), exons (*n* = 3), non-coding RNA (*n* = 1), and TSS region (*n* = 1) of 54 genes. Those genes were selected for further analysis. Next, we compared the distribution of TSS distances between different experimental conditions of shRNA and we observed dynamic changes in regulatory elements activity (Figure 5).

For shjRNA23, we observed an increased number of peaks near TSSs, which can be correlated with repressive elements, MBD2/MBD3-NuRD Complex, that regulate gene expression negatively. TSS distance was greater for shjRNA 23 and shRNA E7, suggesting that MBD2/MBD3-NuRD Complex are enriched at enhancer elements that can regulate gene expression through long-range chromatin interactions. These peaks can still influence transcriptional activity by interacting with the target gene’s promoter region. No peaks for shRNA E7 were found near TSS.

**Figure 6:**
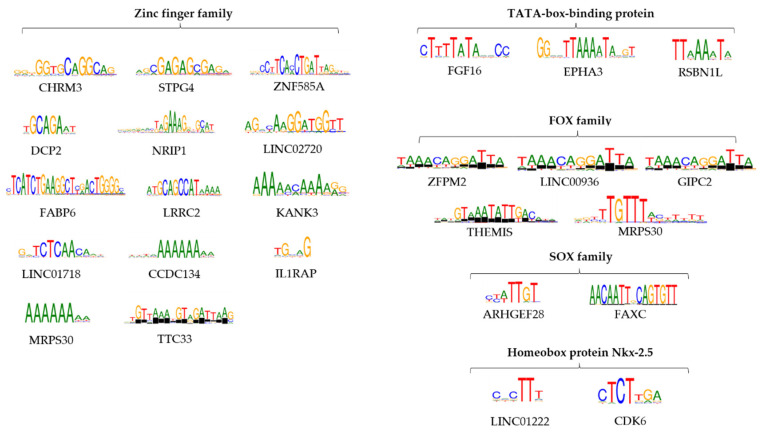
The most important motifs obtained in HOMER analysis and their targets: zinc finger family, TATA-box binding protein, FOX family, SOX family, and Homeobox protein Nkx-2.5.

Differential motif analysis with HOMER was performed to identify short sequences that are over-represented in target sequences compared to background. Based on this analysis, we identified binding sites specific for transcription factors (TFs), regarding the selected genes (Figure 6, Supplementary Table S5). One of the highest scoring recurring motifs is bound to the TATA box binding protein, which was encountered next to the following genes: FGF16, EPHA3, and RSBN1L. Transcription factor IID (TFIID) is an important factor that coordinates the initiation of RNA polymerase II transcription and that binds to the core promoter, positioning the polymerase and serving as a scaffold for complex assembly [20]. Another important mention involves multiple members of the zinc finger family of transcription factors, these motifs ranking among the highest for the following genes: C16orf78, ZNF585A, DCP2, NRIP1, LOC101928446, FABP6, LRRC52, KANK3, LINC01718, CCDC134, and MRPS30. The most common zinc finger members encountered are ZNF384 and ZNF304. Although not the highest scoring, the SOX family of genes was also well represented as a target for the motifs found, with SOX15 and SOX8 as worthy mentions, related to ARHGEF28 (SOX15) and FAXC (SOX8) genes. The SOX (SRY-Box Transcription Factor) family is taking part in the control of embryonic development, proliferation, and migration regulation. There are no reported data regarding the involvement of SOX8 in cervical cancer, but Xie et al. reported a positive association between high SOX8 expression and a high rate of tumor metastasis in tongue squamous cell carcinoma [21]. One other reoccurring family of genes is comprised of forkhead box proteins, which were observed as targets for the motifs next to the following genes: MRPS30 and FAT1–FOXO1; ZFPM2, LINC00936 and GIPC2–FOXJ2; THEMIS–FOXB1. The forkhead family of transcription factors (FOXO) was also noted, being involved in promoting multiple cellular processes such as cycle arrest and apoptosis. ELF3 (E74 Like ETS Transcription Factor 3) is another factor involved in downregulation of DNA-templated transcription and upregulation of transcription by RNA polymerase II. Homeobox protein Nkx-2.5 was also found as a target for motifs encountered for LINC01222 and CDK6. The results highlighted a propensity towards motifs that bind transcription factors involved in key cellular processes such as apoptosis and cell proliferation, immune response and cell cycle arrest, pathways known to be heavily affected in the cancer pathology. These results indicate a much more subtle oncologic process, with many more host gene expressions affected by the HPV16 E6 and E7 oncogenes.

### 3.7. Evaluation of Gene Expression for ChIPseq-Selected Genes in Experimental Model

**Figure 7:**
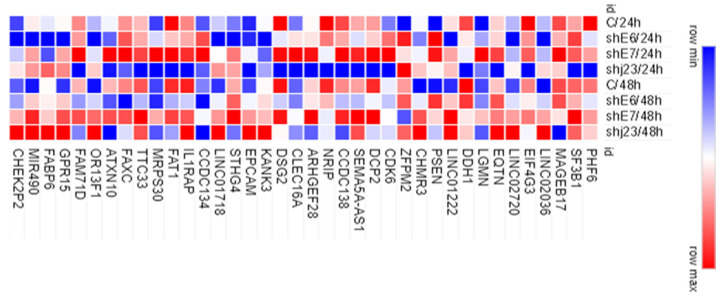
Heatmap of 38 selected genes expression in shRNA experimental model versus control after 24 and 48 h.

For all 54 selected genes, mRNA expression levels were evaluated in shRNA treated versus untreated CaSki cells, through real-time PCR (RT-PCR). Of those, 38 genes were validated at mRNA expression level (Figure 7).

Treatment with shjRNA 23 for 24 h led to a lower expression of the following genes: PHF6, SF3B1, EIF4G3, DCP2, SEMA5A-AS1, CCDC138, NRIP, DSG2, FAT1, and FAM71D both compared to control and shRNA treatments for E6 and E7. Exposure to shRNA E6 for 24 h induced a decrease in the expression of the following genes: LINC02036, LINC02720, LINC01222, LINC01718, miR-490, CHMR3, GPR15, OR13F1, and DSG2. shRNA E7 for 24 h induced the increase of gene expression for: LGMN, CDK6, DCP2, SEMA5A-AS1, CLEC16A, EPCAM-DT, STHG4, MRPS30, and ATXN10. The expression of the following genes did not show significant changes following the treatments used for 24 h: MAGEB17, EQTN, DDH1, KANK3, IL1RAP, TTC33, FAXC, and FABP6. Another category of genes is represented by those with slightly increased expression in all the treatments used (shRNA E6/24 h, shRNA E7/24 h, and shjRNA 23/24 h): PSEN2, ZFPM2, and CCDC134. MAGEB17, ARHGEF28, and STHG4 have a low expression compared to the control after treatment with sh23 for 48 h, an effect that could not be observed within the individual treatments (shRNA E6/48 h and shRNA E7/48 h). In contrast, EIF4G3, CCDC138, and ATXN10 register lower values after treatment with sh23 for 48 h, an effect obtained due to the lack of the E6 oncogene. The genes PHF6, SF3B1, EIFG3, DCP2, SEMA5A-AS1, CCDC138, DSG2, CCDC134, FAT1, TTC33, FAXC, ATXN10, and FAM71D show a more diminished expression profile after treatment with shRNA E6/48 h, which is also reflected in treatment with shjRNA 23/48 h, except SEMA5A-AS1. After treatment with E7 for 48 h, DDH1 and NRIP show a decreased expression, while LGMN, CLEC16A, and MRPS30 have an increased expression. LINC02036, LINC02720, LINC01718, miR490, OR13F1, CHMR3, EPCAM-DT, GPR15, and CHEK2P2 show increased expression as a result of treatment with shRNA E6/48 h and shRNA E7/48 h, with a cumulative effect in the case of shjRNA 23/48. LINC01222 also has an increased expression mainly due to shRNA E7/48 h, being similar to shjRNA 23/48, while PSEN2 has an increased expression profile.

### 3.8. Distribution of HPV Genotypes in Studied Groups

The patient’s samples were categorized into seven groups based on their Papanicolaou test outcomes and the presence or absence of HPV (Table 2). The control group comprised samples categorized as Negative for Intraepithelial Lesion or Malignancy and also negative for HPV (NILM HPV−). Targeted biopsies were performed in selected cases after colposcopy evaluation for further assessment. Importantly, the biopsy findings did not change the classification of the aforementioned cytological groups.

In this study, all enrolled patients underwent HPV testing, revealing that 85.13% (63/74) of the samples tested positive for HPV. Among the NILM samples, HPV infection was detected in 45% (9/20) of cases. From all included samples, HPV16 had the highest prevalence, with 52.38% (33 out of 63) testing positive. Additional high-risk HPV genotypes identified included HPV45, detected in 12.70% (8/63) of samples, and both HPV18 and HPV33, each present in 7.94% (5/63) of samples. The remaining 12 samples exhibited diverse high-risk genotypes, including HPV51, HPV52, HPV53, HPV56, HPV58, or HPV66.

### 3.9. Evaluation of Selected Gene by Real-Time PCR in Patient’s Samples

**Figure 8:**
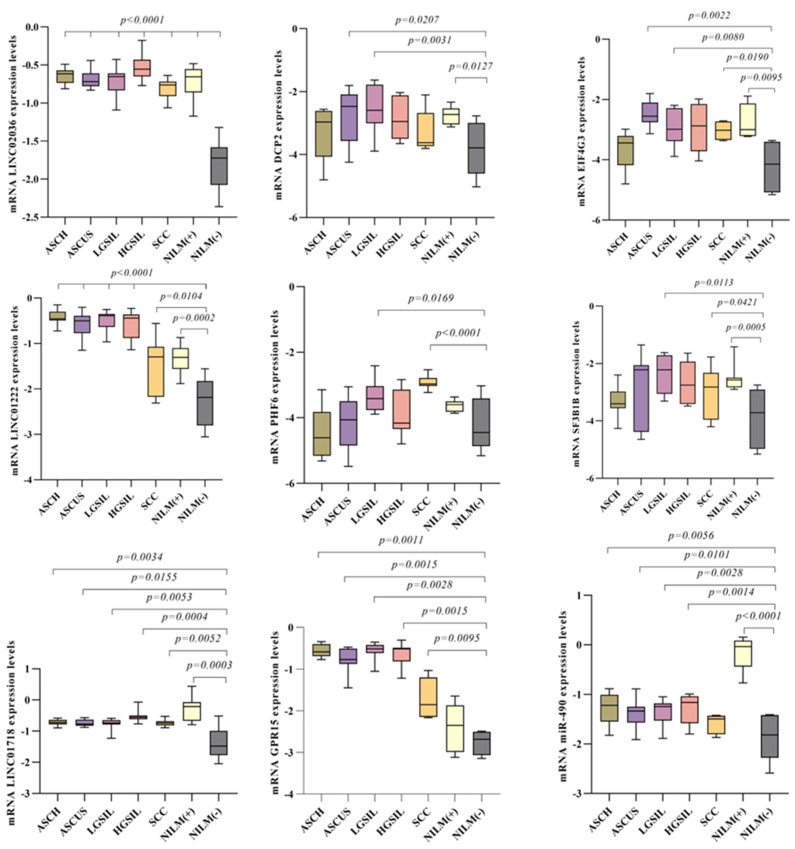
mRNA gene expression levels of selected genes in patient’s samples: LINC02036, DCP2, EIF4G3, LINC01222, PHF6, SF3B1, LINC01718, GPR15, and miR-490.

For this step, we selected the genes with the most significant change at expression levels in treated vs. untreated CaSki cells (PHF6, SF3B1, EIF4G3, DCP2, GPR15, SEMA5A-AS1, CCDC138, NRIP, DSG2, FAT1, FAM71D, ATXN10, miR-490, LINC01222, LINC01718, LINC02036) and evaluated them in the patients’ samples. Our intention was to assess the ability of the selected genes through ChIP-seq to distinguish between cervical precursor lesions, with the goal of determining their potential as biomarkers. The result showed that miR-490, LINC01222, LINC01718, and LINC02036 have significantly increased expression levels in all studied groups compared with the control group. The most significant results were obtained for LINC02036, where all studied groups have statistical increased expression levels compared with NILM(−) (*p* < 0.0001). For miR-490, the most significantly increased expression was observed in NILM(+) (median = −0.036; *p* < 0.0001) versus NILM(−) (median = −1.819). Moreover, LINC02036, LINC01222, and LINC01718 have similar expression patterns. EIF4G3 and GPR15 are other genes that exhibited an increased level of expression in all studied groups, except the ASCUS group and NILM(+). The SF3B1 gene expression pattern revealed increased levels in LGSIL (median = −2.220; *p* = 0.0113), SCC (median = −2.820; *p* = 0.0421), and NILM(+) (median = −2.574; *p* = 0.0005) groups when compared with control (median = −3.719). A similar pattern was observed in the PHF6 expression gene in LGSIL (median = −3.414; *p* = 0.0169) and SCC (median = −2.970; *p* < 0.0001) groups versus NILM(−) (median = −4.451). For DCP2 gene, significant results were observed in ASCUS (median = −2.961; *p* = 0.0207), LGSIL (median = −2.595; *p* = 0.0031), and NILM(+) (median = −2.724; *p* = 0.0127) groups. All results are displayed in Figure 8.

### 3.10. Canonical Pathways, Disease and Functions, Networks

**Figure 9:**
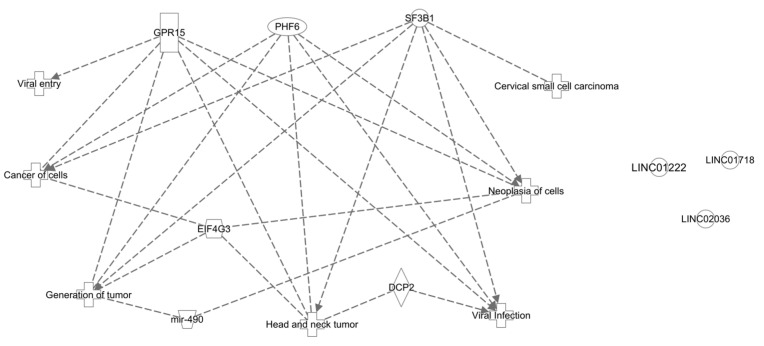
Graphical representation of “Disease and Functions” analysis for selected genes.

To create a custom pathway, we included in our dataset list the following genes: PHF6, SF3B1, EIF4G3, GPR15, DCP2, miR-490, LINC01222, LINC01718, and LINC02036. When building the network, it was observed that all genes were identified in the QKB database, except the LINCs. When running the “Grow to Disease and Functions” feature, 163 most significant diseases and functions resulted. From those, we selected the most important ones in cancer oncogenesis, viral infection, or squamous cell carcinoma context (Figure 9).

**Figure 10:**
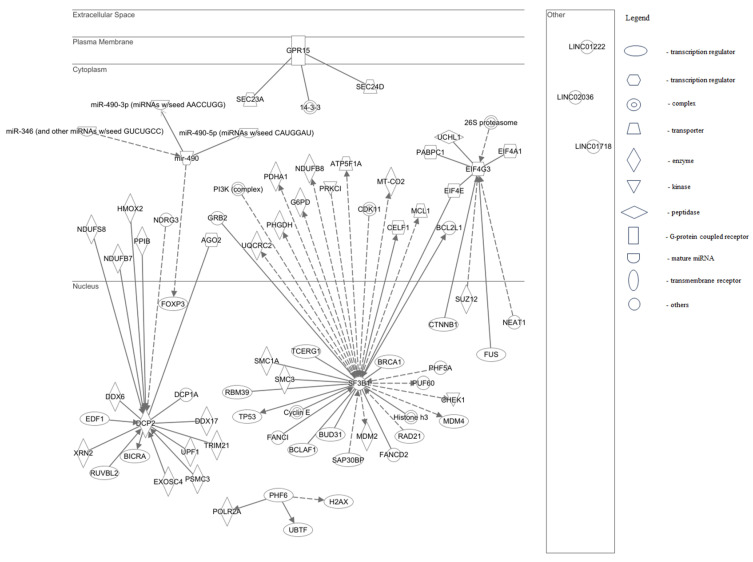
Cellular compartments localization and molecular interaction for proteins encoded by selected genes. The sharp arrowheads symbolize active relationships, whereas the dashed lines denote virtual relationships that encompass the overall impact of the pathways between the primary regulator and the target genes.

When performing the "Grow to Canonical Pathways” tool, we selected only molecules or pathways that include “Cancer”, together with “Infectious Disease”, “Reproductive Disease System” diseases, and “Human” species. The results displayed 38 molecules for the SF3B1 protein with localization in cytoplasm (n = 18) and nucleus (n = 20). The molecules were classified according to their function in: transcription regulators (TP53, BRCA1, MDM31, BCLAF1, SAP30BP, BUD31, RAD21, RBM39, TCERG1), translational regulators (EIF4E, CELF1), enzymes (SMC1A, SMC3, MDM2, PHGDH, UQCRC2, PDHA1, NDUFB8, MT-CO2, G6PD), transporters (ATP5F1A, MCL1), kinases (PRKCI, CHEK1), complexes (PI3K complex, Histone.h3, CDK11, Cyclin E), and others (GRB2, BCL2L1, FANCD2, FANCI, PHF5A, PUF60). For the DCP2 gene, 17 molecules with localization in cytoplasm (n = 6) and nucleus (n = 11) were displayed. Their functions were classified in: enzymes (DDX6, DDX17, PSMC3, EXOSC4, TRIM21, UPF1, XRN2, NDUFB7, NDUFS8, PPIB, HMOX2), transcription regulators (BICRA, EDF1, RUVBL2), translation regulator (AGO2), and others (DCP1A, NDRG3). Of the eight molecules found for the EIF4G3 protein, four were located in cytoplasm and four in the nucleus. Their categorization based on their function was: two translational regulators (EIF4A1, PABPC1), two transcription regulators (FUS, CTNNB1), and one of the following categories: enzyme (SUZ12), complex (26S proteasome), peptidase (UCHL1), and other (NEAT1). For PHF6 and GPR15, proteins were displayed three molecules each. The PHF6 ones were found in the nucleus, two of them with the function of transcription regulators (H2AX, UBTF) and one as an enzyme (POLR2A). On the contrary, the GPR15 ones were found in cytoplasm, two of them being transporters (SEC23A, SEC24D) and one being a complex (14-3-3). Of the four targets of miR-490, three of them are located in cytoplasm, and as a function they are mature microRNA (miR-490-3p, miR-490-5p and miR-346), while one was located in nucleus, being a transcription regulator (FOXP3). No molecules were found for the other genes. We also observed that EIF4E is a common target for both EIF4G3 and SF3B1 factors (Figure 10).

**Figure 11:**
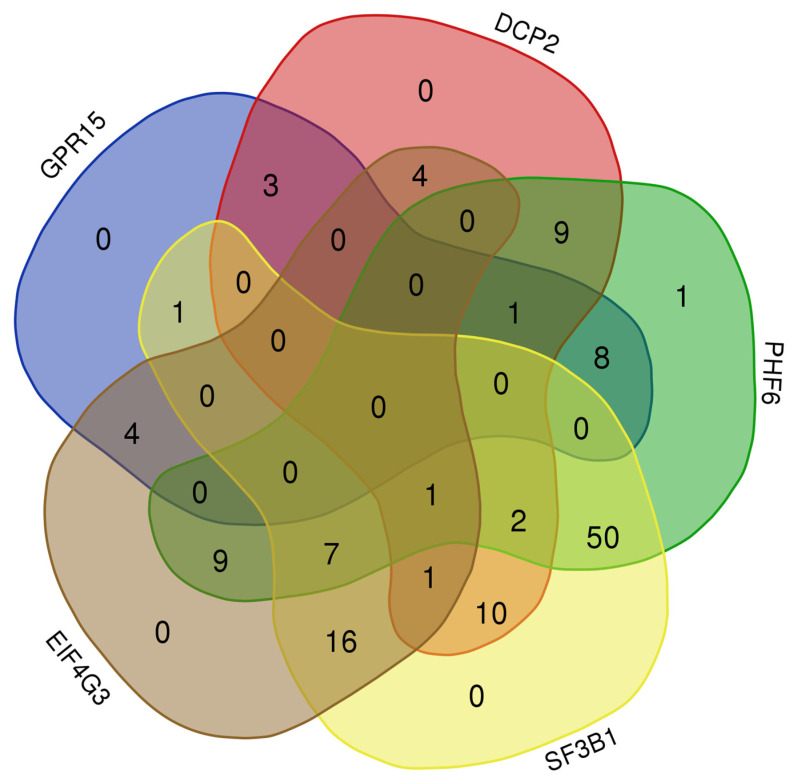
Venn Diagram showing the overlap between targeted molecules of DCP2 (30), EIF4G3 (42), PHF6 (88), SF3B1 (88), and GPR15 (17).

Using the “Path Explorer” tool helped identify the shortest path between inserted nodes. We used this tool to build up custom literature-supported signaling pathways starting with the same set of genes, when defining in the set-up menu Set A and Set B. Moreover, we selected the same parameters to include “Cancer”, “Infectious Disease”, and “Reproductive Disease System” diseases. Thus, we obtained 265 pathways that linked the molecules between them. The pathways were classified according to their type of relationship in: 187 protein–protein interaction, 7 protein–RNA interaction, and 71 RNA–RNA interaction (microRNA targeting). We designed a Venn diagram that illustrates the number of overlapping and distinct factors. The most common factors were found between PHF6 and SF3B1 proteins (n = 50), followed by SF3B1 and EIF4G3 (n = 16). When intersecting PHF6, SF3B1, and EIF4G3 proteins, seven common targets were observed (MYCN, NAA40, NTRK1, PRKN, STAU1, TRIM67, ESR1). Only one target, PLEKHA4, was displayed after intersecting DCP2, EIF4G3, PHF6, and SF3B1 proteins. No common factors were found when overlapping all five proteins (Figure 11).

Applying again the “Build”–“Grow to Diseases and Functions” feature, we obtain a list of 437 most important diseases and functions. The most significant ones were “Solid tumors” and “Malignant solid tumor”, which involved 109 and 108 molecules, respectively, from a total of 111 molecules. As we have seen in HOMER analysis, more forkhead box protein family members were found to be targets for various motifs. Interestingly, we observed that Foxp3 was a target for miR490. Foxp3 and p16INK4a expressions elevate as cervical lesions progress, with Foxp3 expression being positively associated with p16INK4a expression in cervical cancer. This indicates that the increased expression of Foxp3 and p16INK4a may contribute as one of the mechanisms driving the occurrence and progression of cervical cancer [22].

When performing “Overlay”–“Disease and Function” feature, we observed that the PHF6 protein and associated molecules lead to the activation of canonical pathways involved in: expression of RNA along with 45 other targets such as: MYC, EZH2, SUZ12, and SOX2 (*p* = 1.23 × 10^−26^), activation of DNA endogenous promoter with other 33 molecules (*p* = 4.54 × 10^−25^) and transcription together with 41 factors. Chang et al. observed a significant overexpression of SOX2 in cervical squamous cell carcinoma (SCC) samples compared to normal cervical tissue [23].

Concerning SF3B1, our investigation revealed its participation in the processing of capped intron-containing pre-mRNA, alongside seven other molecules including POLR2A and PAF5A. Likewise, our findings indicate that DCP2 is engaged in deadenylation-dependent mRNA decay, in conjunction with six other molecules such as DCP1A, EIF4E, and EIF4A1. Additionally, GPR15 was identified to be implicated in the molecular mechanism of cancer, together with 11 molecules including TP53, BRCA1, and Cyclin E, and also in FAK signaling and S100 Family signaling pathway.

Next, we added NuRD complex as a new molecule and ran the “Path Explorer” tool between SET A (NuRD) and SET B (SF3B1B, PHF6, EIF4G3, DCP2, GPR15, miR-490, LINC01222, LINC01718, LINC02036) with the same parameters for diseases: “Cancer”, “Infectious Disease”, and “Reproductive Disease System”. The findings unveiled 11 nodes linking the NuRD complex to three out of nine genes (SF3B1B, PHF6, and EIF4G3). Including these molecules resulted in the identification of 19 pathways within the network.

## 4. Discussion

Hr-HPV displays its transforming properties through E6 and E7 interaction with host cell components. The NuRD complex, as a distinctive group of proteins responsible for both chromatin remodeling and histone deacetylation, is such a target for the viral oncoproteins [24,25]. NuRD-mediated deacetylation of histone H3K27 specifies the recruitment of PRC2 in embryonic stem (ES) cells. Such deacetylation promotes PRC2 recruitment and the subsequent H3K27 trimethylation at NuRD target promoters. Hence, the knockdown of the NuRD complex results in the deregulation of several bivalent genes, increasing H3K27ac and reducing H3K27me3 as well [26]. Chromatin immunoprecipitation followed by next-generation sequencing (ChIP-Seq) is the most relevant technique in viral chromatin investigation, examining binding patterns of modified histones, transcription factors, or other DNA-/chromatin-binding proteins that regulate the viral life cycle [27,28]. In our study, ChIP-seq analysis established the most important targets of the MBD2/MBD3 NuRD complex by silencing E6 and E7 oncogene expressions. ChIP-seq data showed that treatment with shjRNA 23 in our experimental model determined an increased number of peaks near TSSs correlated with repressive elements and with enhancer elements regulating gene expression. Our results identified several genes whose transcriptional activity is influenced by HPV16 E6/7 viral oncogenes through the MBD3/MBD3 NuRD complex. Targeting both viral oncogenes by shjRNA 23 seems to have an important impact on gene expression transcription and apoptosis. Blocking both the E6 and E7 oncogenes leads to a greater percentage of cells entering the apoptotic process compared to when either E6 or E7 oncogenes are inhibited individually. Differential motif analysis indicated binding sites specifically for transcription factors (TFs), concerning genes involved in pathways frequently disrupted in cancer as transcription regulation, diverse biological processes such as cell differentiation, proliferation, immune response, apoptosis, cell cycle arrest, and oxidative stress responses. One of the genes with modified expression patterns by HPV16 viral oncogenes through MBD3/MBD3 NuRD complex chromatin deposition is PHF6, which encodes for a chromatin-bound protein. This protein plays a multidimensional role in gene regulation by recognizing histone methylation marks, interacting with protein complexes such as NuRD and BRWD2/PHIP, and controlling various aspects of transcription, cell cycle, and the cross talk between histone acetylation and methylation [29]. Despite being recognized as a tumor suppressor, it was found overexpressed in various cancer tumors, which may indicate an oncogenic role in these types of cancer. Furthermore, proteomic analysis reveals high expression levels of PHF6 in cancerous tissues such as lymphoma, glioma, colorectal, and cervical cancer [30,31]. Our findings also revealed that the PHF6 gene exhibited decreased expression in shjRNA 23 in an experimental model and significantly increased expression levels in SCC and LGSIL groups. PHF6 transcription seems to be activated by E6 and E7 HPV16 viral proteins. The PHF6 protein and associated molecules lead to the activation of canonical pathways involved in expression of RNA along with 45 other molecules. Besides *PHF6*, shjRNA 23 treatment in the experimental model led to a lower expression of the following genes: *SF3B1*, *EIF4G3*, *DCP2*, *SEMA5A-AS1*, *CCDC138*, *NRIP*, *DSG2*, *FAT1*, and *FAM71D*. Evaluation of the SF3B1 gene expression pattern revealed increased levels in precursor lesions and SCC, while for the DCP2 gene, significant results were observed in ASCUS, LGSIL, and NILM (+). SF3B1 could be a potential biomarker for cervical cancer, while DCP2 may serve as a viral infection indicator. SF3B1 is responsible for encoding the primary subunit found in the spliceosome factor 3b (SF3B) complex, which plays a pivotal role within spliceosomes, essential molecular machinery for RNA processing. Numerous studies have highlighted the role of SF3B1 overexpression in promoting the development of various cancers such as breast cancer, prostate cancer, and myelodysplastic syndromes [32,33]. Furthermore, a recent investigation demonstrated that suppressing the elevated levels of SF3B1 significantly decreased breast cancer cell proliferation, migration, and invasion [34]. Elevated levels of SF3B1 protein expression have been observed in human endometrial tumors as well as three different endometrial cancer cell lines. This finding aligns with numerous previous studies that have reported increased expression of various other splicing factors in a variety of human cancers [35].

Decapping mRNA 2 (DCP2) enzyme belongs to the Nudix superfamily of hydrolase proteins that primarily functions in catalyzing the hydrolysis of various small nucleotide substrates, where a nucleoside diphosphate is linked to an additional moiety X [36,37]. The expression of DCP2, a protein involved in gene regulation, is detected in almost all tissues during embryonic development in mice. However, it shows high expression levels specifically in the testis and brain, moderate expression in the spleen and lung, and is not detected in the heart, liver, kidney, and muscle tissues of adult mice [38]. This indicates that DCP2 plays a role in regulating gene expression in a context-dependent manner, varying both temporally and spatially.

GPR15 presented a decreased gene expression in shRNA E6 treatment and presented an increased level of expression in all patient groups (including NILM+) compared to control, being correlated with cervical oncogenesis and viral infection. GPR15, also referred to as BOB, is a seven-transmembrane domain, class A G-protein coupled receptor (GPCR). Initially recognized as a co-receptor for human immunodeficiency virus (HIV) or simian immunodeficiency virus (SIV), GPR15 is expressed on regulatory and effector CD4+ T cells, fetal thymic epidermal T cells, B cells, and plasma blasts [39,40]. GPR15 exhibits high expression in tissues interfacing with the external environment, such as the skin and mucosal epithelium, crucial for maintaining immune cell residence and immune barrier function. Consequently, overexpression of GPR15 is easily comprehensible in skin and intestinal inflammation conditions like psoriasis, atopic dermatitis, and lichen planus. [41,42].

EIF4G3 also presented a higher expression in precursor lesions compared with control samples. EIF4G3 is known as eukaryotic translation initiation factor 4 γ 3, a protein involved in the process of translation initiation in eukaryotic cells. It is a component of the EIF4F complex, which plays a crucial role in the initiation of protein synthesis by binding to the mRNA cap structure and recruiting the ribosome to the mRNA. Besides its role in translation initiation, EIF4G has recently been suggested to have alternative functions such as involvement in nuclear mRNA biogenesis and surveillance [43].

The genomic locus encoding miR-490 is situated within the intronic region, specifically the second intron, of its host gene Cholinergic Receptor Muscarinic 2 (CHRM2) located on Chromosome 7. This locus is highly conserved across species and encompasses eight transcript variants of CHRM2, an uncharacterized long noncoding RNA (LOC349160), and two mature miRNAs, miR-490-3p and miR-490-5p, alongside a keratin 8 pseudogene 51. Both miR-490 and CHRM2 display coordinated expression, with the highest levels observed in the heart, followed by the gall bladder and urinary bladder [44]. Our study showed similar results with Ding et al., who reported up-regulation of miR-490 in tumor tissues/plasma in cervical cancer [45].

One of the most important observations was that the shRNA E6 treatment induces a decrease in the expression of the following genes: *LINC02036*, *LINC01222*, *LINC01718*, *miR-490.* These genes presented a significantly increased expression level in all studied patients groups compared with the control group, being a potential marker for hrHPV infection. Moreover, for all LINCs identified the function is not fully elucidated, this study being the first mentioning the involvement in cervical oncogenesis.

Canonical pathway analysis revealed elements categorized as transcription regulators, translational regulators, enzymes, transporters, and kinases, involved in cancer oncogenesis, viral infection, or squamous cell carcinoma scenarios. The DCP2 protein engages with factors situated in both the cytosol and nucleus, playing significant roles in transcription and translation regulation. EIF4G3 protein was observed to interact with both translational and transcriptional regulators. Three molecules were associated with each of the PHF6 and GPR15 genes. Additionally, PHF6 and miR-490 also interact with transcription regulators. Our data showed that PHF6 protein and associated molecules lead to the activation of canonical pathways involved in expression of RNA and cancer progression by interaction with several oncogenes.

## 5. Conclusions

Our study demonstrated that the E6 and E7 HPV16 oncogenes have the capability to alter the global chromatin deposition of the MBD2/MBD3 NuRD complex. Through our investigation, we identified several factors associated with viral infection and cervical oncogenesis. Some of these analyzed factors were confirmed in patients, suggesting their potential as prognostic biomarkers. The discovery of motifs for transcriptional factors may pave the way for exploring novel targets for the treatment of cervical cancer and HPV infection. Our findings indicate that E6/E7 HPV16 viral proteins participate in the regulation of gene transcription, mRNA processing, and translation regulation. Unveiling such pathways provides crucial insights into understanding the process of HPV-induced oncogenesis.

## Figures and Tables

**Table 1:** The specific nucleotide sequences targeted within the full-length HPV16 E6 and E7 transcripts.

shRNA	Targeted Nucleotide Sequence in Full-Length E6 and E7 HPV16 Transcript	shRNA Nucleotide Sequence (5′-3′)
shRNA-1	272–292 E6	GGGAATCCATATGCTGTATGT
shRNA-2	503–523 E6^E7 joint	GGTCGATGTATGTCTTGTTGC
shRNA-3	189–209 E6	AATGTGTGTACTGCAAGCAAC
shRNA-4	702–722 E7	GGACAGAGCCCATTACAATAT
shRNA-5	709–729 E7	GCCCATTACAATATTGTAACC

**Table 2:** The classification of patients’ samples according to the cytological analysis, HPV status and age. NILM (Negative for Intraepithelial Lesion or Malignancy) positive and negative; ASCUS (Atypical Squamous Cells of Undetermined Significance); ASCH (Atypical Squamous Cells); LGSIL (Low-Grade Squamous Intraepithelial Lesion); HGSIL (High-Grade Squamous Intraepithelial Lesion); SCC (squamous cervical carcinomas).

	Total Number of Cases, *n* = 74 (100%)						
	NILM HPV−	NILM HPV+	ASCUS HPV+	LGSIL HPV+	ASCH HPV+	HGSIL HPV+	SCC HPV+
Number of cases	*n* = 11	*n* = 9	*n* = 12	*n* = 12	*n* = 11	*n* = 10	*n* = 9
(% from total number of cases)	(14.86%)	(12.16%)	(16.21%)	(16.21%)	(14.86%)	(13.51%)	(12.16%)
Age (Mean ± SD)	33.88 ± 4.224	36.22 ± 7.496	32.00 ± 6.663	31.45 ± 8.042	36.00 ± 8.790	37.13 ± 9.062	62.44 ± 14.910

## Data Availability

The original contributions presented in the study are included in the article and Supplementary Material, further inquiries can be directed to the corresponding author.

## Supplementary Materials

The following supporting information can be downloaded at: https://www.mdpi.com/article/10.3390/genes15050560/s1; Table S1: Primer sequences for the investigated target genes and their silencing efficiency at three concentrations: C1, C2, and C3, after 24 and 48 h. Table S2: Primer sequences for the investigated target genes; Table S3: Cell cycle and total apoptosis distribution in silenced or unsilenced CaSki cells with shRNAs; Table S4: Time dependent percentage of silencing with shRNAs; Table S5: Significantly enriched motifs and associated transcription factors. Table S6. Common molecular targets for identified factors. Figure S1: Flow cytometry histograms representing treated versus untreated CaSki cells in G1, G2+M and S phases; Figure S2: Quality control—"Phantom peaks" histograms in shRNAs ChIP—Seq samples.
