# Supplementary material for: Exploring the Role of E6 and E7 Oncoproteins in Cervical Oncogenesis through MBD2/3-NuRD Complex Chromatin Remodeling
Source: Genes (Basel). 2024 Apr 27;15(5):560. doi: 10.3390/genes15050560 (PMC11121560; doi:10.3390/genes15050560)
Supplement: Supplementary file 1 [file genes-15-00560-s001.zip › Supplementary tables.pdf]

Supplementary Tabel S1. Primer sequences for the investigated target genes and their silencing efficiency at three concentrations: C1, C2 and C3, after 24, respectively 48 hours.

**shRNA name**	**Sh oligonucleotides sequences**	**shRNAs concentrations an efficiency (%)**					
		C1-4μg		C2-6μg		C3-8μg	
		24h	48h	24h	48h	24h	48h
shE6RNA_208_top	CACCGGGAATCCATATGCTGTATGTCGAAACATACAGCATATGGATTCCC						
shE6RNA_208_bottom	AAAAGGGAATCCATATGCTGTATGTTTCGACATACAGCATATGGATTCCC	68.83%	43.72%	80,45%	55.71%	75,01%	51,02%
shE6RNA_439_top	CACCGGTCGATGTATGTCTTGTTGCCGAAGCAACAAGACATACATCGACC						
shE6RNA_439_bottom	AAAAGGTCGATGTATGTCTTGTTGCTTCGGCAACAAGACATACATCGACC	60.11%	41.85%	75.98%	42.45%	68.11%	44,25%
shjRNA_23_top	CACCGCTGTAATCATGCATGGAGATCGAAATCTCCATGCATGATTACAGC						
shjRNA_23_bottom	AAAAGCTGTAATCATGCATGGAGATTTCGATCTCCATGCATGATTACAGC	74.53% E6<br>50.35% E7	50.45% E6<br>30,27% E7	79.51% E6<br>66.66% E7	43.18% E6<br>30.26% E7	63.04% E6<br>47.75% E7	69,34% E6<br>31.33% E7
shE7RNA_158_top	CACCGGACAGAGCCCATTACAATATCGAAATATTGTAATGGGCTCTGTCC						
shE7RNA_158_bottom	AAAAGGACAGAGCCCATTACAATATTTCGATATTGTAATGGGCTCTGTCC	55.01%	43.80%	65.27%	49,83%	63.31%	39.81%
shE7RNA_165_top	CACCGCCCATTACAATATTGTAACCCGAAGGTTACAATATTGTAATGGGC						
shE7RNA_165_bottom	AAAAGCCCATTACAATATTGTAACCTTCGGGTTACAATATTGTAATGGGC	53.9%	35.82%	55,81%	44,17%	59.81%	41.74%

Supplementary Tabel S2. Primer sequences for the investigated target genes

Gene	Primer forward	Primer reverse
**E6HPV16**	5′-GCATAAATCCCGAAAAGCAA-3′	5′-AGCGACCCAGAAAGTTACCA-3′
**E7HPV16**	5′-GCTCAGAGGAGGAGGATGAAATAG-3	5′-TCCGGTTCTGCTTGTCCAG-3′
**GAPDH**	5′-CCATCTTCCAGGAGCGAGATCCCT-3′	5′-TGAGCCCCAGCCTTCTTCATGGT-3′
**PHF6**	5′-GGAGAACCGAGACCGACTTC-3′	5′-GTCTCAAGAAATGCGGCAGG-3′
**SF3B1**	5′-AAAAGCATAGGCGGACCATGA-3′	5′-GGGGTTTTCCCTCCATCTGC-3′
**FGF16**	5′-CTGGAGATCTTCCCCAACGG-3′	5′-AACTCCAGGATTCCGAAGCG-3′
**MAGEB17**	5′-CCGGAAGTGAGGATCTTCGT-3′	5′-GGAGGTGGGCACCTTGATAC-3′
**LINC02036**	5′-AGCTCACCGGTAGCCTAGAA-3′	5′-ATATGGCTCAGCCGCATCTC-3′
**EIF4G3**	5′-ACAGAATGCAGGTCCAACCA-3′	5′-GGCCTCTGGAAAAACGGAGA-3′
**LINC02720**	5′-AGCTCACCGGTAGCCTAGAA-3′	5′-ATATGGCTCAGCCGCATCTC-3′
**EQTN**	5′-TCCAAATGGCACTGAGTCTGA-3′	5′-AGTTGCATTGACAGTTTTATCGTT-3′
**KCNJ3**	5′-CTCACGCTTATGTTCCGGGT-3′	5′-GTGTCTGCCGAGATTTGAGC-3′
**LGMN**	5′-CACAGCTGCTACCCAGAGG-3′	5′-AACGCATACTCGTACGTGGG-3′
**DDHD1**	5′-ATTGTTTTAGGGGCCAGCAGA-3′	5′-AGTTGATCCCACTGCCATCT-3′
**LINC01222**	5′-AGCAGGGGTAACATTATGGGC-3′	5′-AGTCTTTGTAGGTCACCAACCC-3′
**PSEN2**	5′-AAGGGCCTCTGAGAATGCTG-3′	5′-CCACACCATGGCAGATGAGT-3′
**CHMR3**	5′-CTACCTGGAACAGGCCAACAC-3′	5′-TGCCAATAAGACTTGTGAGTTCCA-3′
**ZFPM2**	5′-TCCACAGAATTTGGGCCTGA-3′	5′-TGGATTCCTTCATCATCACCTTTG-3′
**THEMIS**	5′-CTGCCTATGAATTTTCCAGGTCT-3′	5′-TGGTCCAATATGAATGGTCCTTGT-3′
**CDK6**	5′-CTGCAGGGAAAGAAAAGTGCAA-3′	5′-CTCCTCGAAGCGAAGTCCTC-3′
**TRIM60**	5′-TCCAACTGCTCCAGGTCATC-3′	5′-AGGTTATAATACAGCGGTCATCCC-3′
**LINC00936**	5′-GCGTTTAAGGGGAGGACCC-3′	5′-GCGCGGCAAGTTCTAATCG-3′
**GIPC2**	5′-TCTCGCCGTCGGAGATCTTA-3′	5′-CCTCCTAAGAGTCTTTCCATGTCA-3′
**DCP2**	5′-GGTAAAGCACAGGCAACCAC-3′	5′-TCCCCTCATACTTTGATTCTTTCCT-3′
**DLC1**	5′-GCTCCTCGTTGTCTAGGTGG-3′	5′-TGAGGGTAAAGGAGATGGAACT-3′
**SEMA5A-AS1**	5′-CCTTGTGGCCACTACTGAACT-3′	5′-GTGGCATTCCCCATAGGCTT-3′
**CCDC138**	5′-CTGCCCCGACGAGTATGATT-3′	5′-CCAAATCACCTGGGGAGGTT-3′
**NRIP1**	5′-ACACAGCCAGAAGATGCACA-3′	5′-AGGCTGTTGAAAAGTAGCTCTGA-3′
**ARHGEF28**	5′-CCATCGCTCCAGATGCGAAA-3′	5′-GCATAGATCATCATCTGCCCGT-3′
**CLEC16A**	5′-CAACCACCCTGAAAGCATGG-3′	5′-GGCCTGGTTATCCAATGACAC-3′
**CDH17**	5′-ATCGACCCACGTTTCTCCAG-3′	5′-CAGTCCTCTCTAGGAGATGAAGTTT-3′
**ATXN10**	5′-AGAGCAGCGGAACCGAGAA-3′	5′-GCAGGCAAGCTCAACAGCAT-3′
**EPHA3**	5′-TCACGGGTGTGGAGTACAGT-3′	5′-ACCAACCTTTTTCATGTCATCTGT-3′
**LRRC4C**	5′-GTAGTGCACGGCAAGCTAGA-3′	5′-TCGCAGTAAAGAGAACCATTTCTTC-3′
**OR13F1**	5′-TGGTTTGTGTGGACACCTCC-3′	5′-TGAGTAGCATTGGCATGGGG-3′

**FAM71D**	5′-AGCCCCACCTGTTGATTTCAT-3′	5′-CAGTCCTCTCTAGGAGATGAAGTTT-3′
**LOC101928446**	5′-CCCAGGGATCCCCTGACATA-3′	5′-GAACCTGGGTGGGCCTTATT-3′
**PTGDR**	5′-GGGAGTAGGTGAGGCTTGAG-3′	5′-GTAAGCGCGATACTTGGGAG-3′
**GPR15**	5′-TGCTCTTTGGTGATGGACCC-3′	5′-CAGAGTTTGGGCTCGTAGCA-3′
**FABP6**	5′-GCCACACCATGACCAACAAG-3′	5′-ATCTGCACAGTGGCCTTGAA-3′
**MIR490**	5′-TGGAGGCCTTGCTGGTTTG-3′	5′-TCCTCCAGGTTGGTGCATCT-3′
**LRRC52**	5′-TGCTCTTTCCTGGACTTCGC-3′	5′-TGGCATTTAGATCATCTGAGGGG-3′
**CHEK2P2**	5′-TGGTAGTGGATCCAAAGGCAT-3′	5′-GTCTTCATCCTGAAGCCACAGT-3′
**DSG2**	5′-TGCTGCTTCTCCTGATCTGC-3′	5′-GCAGCTTATTTCATTTCTTGTGCT-3′
**KANK3**	5′-TGCGGCTTCAGGGTGC-3′	5′-GCAGGTTCTGATTCAGGGCA-3′
**EPCAM-DT**	TGGGGGATGAAGGGTGGTTA-3′	TTCCTGGCATATCAGGGGGA-3′
**STHG4**	GCCCAGCTGTTCAAAAACCC-3′	GCCCATTTTGGCTATGGTCG-3′
**PROKR2**	GTGACTCTCGATCCGGCTTC-3′	GCTGCCATGGTGATGTCTGT-3′
**LINC01718**	CTCCACAGCCTCGTGTGTTA-3′	TCGCTATGATTTCCTCCGGC-3′
**CCDC134**	GCTGTTAGCCTGTTGGACCT-3′	TCCAAACCTCTTGAGCTGGC-3′
**IL1RAP**	ACTAGAACATCAGCAGGCCC-3′	ACCACAGAAGTGTCATCCTTTGA-3′
**FAT1**	CGGGCCAACTTTGCGATTC-3′	GGAGCAGAAGCAGGAGCAAA-3′
**RHOH**	TTCTCCTTCACACACCAGTTGA-3′	CTTCTCCCTGCCCATCCAAG-3′
**MRPS30**	ATCAGCTGGTGTCAACCCTC-3′	GGGCATCTATAATCGAGGGCG-3′
**TTC33**	CCGCTTCTGTGGACTGTTCG-3′	ACCCAAAGGAAGCCATTCTGC-3′
**FAXC**	ACTCCTGGTCATTAGGAAACAGC-3′	GGAACACCATTGTTAGGTCTTGC-3′

Supplementary Table S3: Cell cycle and total apoptosis distribution in silenced or unsilenced CaSki cells with shRNAs

shRNAs	**24 hours**				**48 hours**			
				Total apoptosis				Total apoptosis
	**%G1**	**%G2+M**	**% S**	**Q2+Q4**	**%G1**	**%G2+M**	**% S**	**Q2+Q4**
**shRNAE6**	62.6	1.8	35.5	1,7+ 14,9 = 16,6%	53.1	0.3	46.6	10,7 +42=52,7%
**shRNAE7**	62.6	0.3	37.1	12,8+ 2,5=15,3%	56	4	40	24,2 +12,9=37,1%
**shjRNA23**	57.3	2.5	40.2	10,2+ 22,3=32,5%	69.4	17.5	14.1	19 +48,2=67,2%
**Control**	55	10.8	34.2	1.5+0.3=1.8%	53.5	0.5	46	8.5+1.3=9.8%

Supplementary Table S4: Time dependent percentage of silencing with shRNAs

**shRNA**		**Percentage (%) of silencing / 24 hours**	**Percentage (%) of silencing / 48 hours**
**shjRNA23**	**E6**	79.51	43.18
	**E7**	66.66	30.26
**shE6RNA**		80.45	55.71
**shE7RNA**		63.27	49.83

Table S5. Significantly enriched motifs and associated transcription factors

**Genes**	**Motifs**	**Stamp E-value**	**Transcriptional factors**
	**shE6RNA**		
MAGEB17	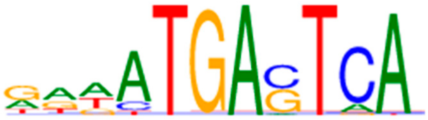	2.5e-09	BATF::JUN
LINC02036	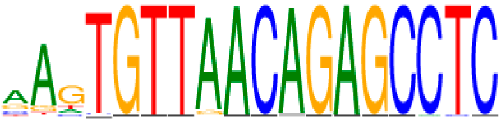	2.1e-09	ZCSAN16
EIF4G3	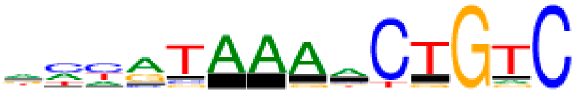	2.4e-09	HOXA13_MEIS1
LINC01222	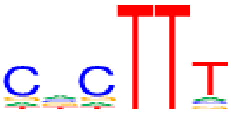	3.7e-09	Homeobox protein Nkx-2.5
PSEN2	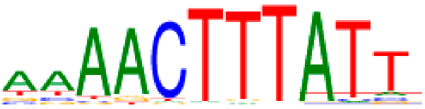	2.2e-08	ZBTB40
CHRM3	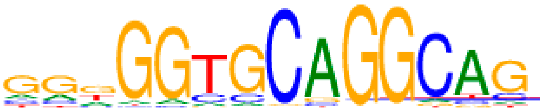	2.8e-08	ZNF223
	**shE7RNA**		
FGF16	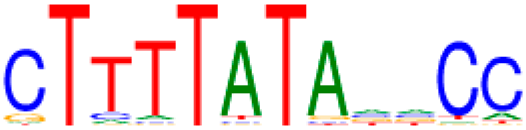	1.8e-09	TATA box binding protein
PHF6	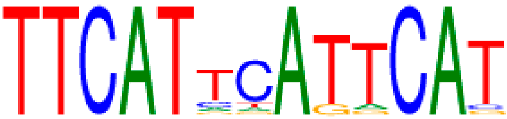	1.3e-07	Pituitary-specific positive transcription factor 1
SF3B1	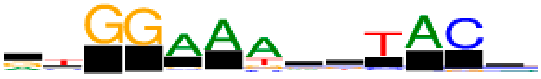	2.2e-09	NFAT5

OR13F1	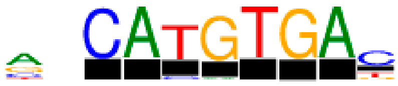	3.9e-10	TFEB
EPHA3	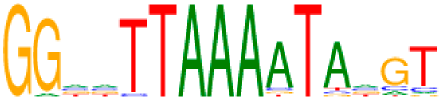	2.0e-07	TATA-box-binding protein
ATXN10	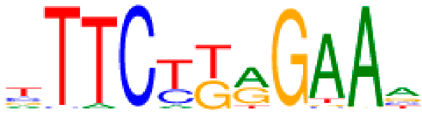	1.1e-07	STAT5B
LRRC4C	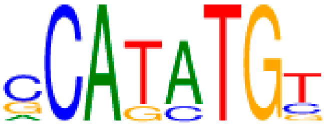	8.9e-10	Bhlha15
RSBN1L	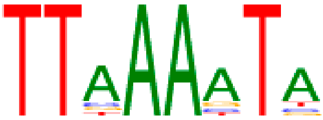	7.8e-09	TATA-box-binding protein
MRPS30	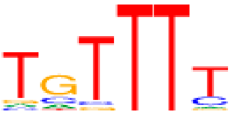	6.5e-09	Forkhead box protein O1
C16orf78 (STPG4)	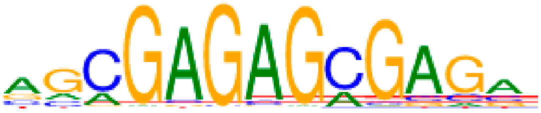	1.9e-08	ZNF596
ZNF585A	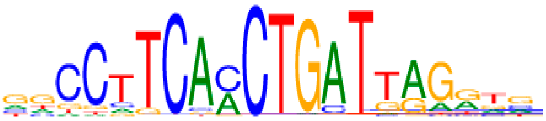	9.4e-07	ZNF134
**shj23RNA**			
ZFPM2	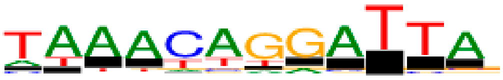	4.8e-07	FOXJ2_PITX1, FOXJ2::PITX1
THEMIS	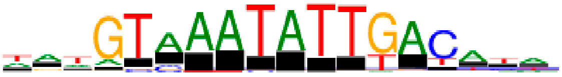	9.4e-06	FOXB1
CDK6	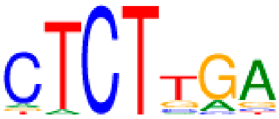	2.2e-07	Homeobox protein Nkx-2.5

TRIM60	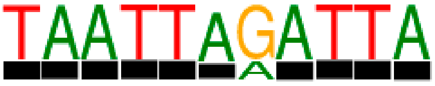	3.4e-08	ALX1
LINC00936	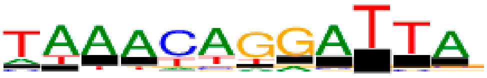	1.3e-07	FOXJ2_PITX1, FOXJ2::PITX1
GIPC2	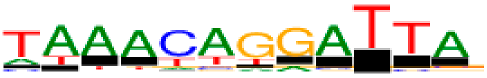	1.8e-06	FOXJ2_PITX1, FOXJ2::PITX1
DCP2	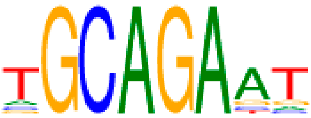	6.4e-07	Zinc finger protein 217
DLC1	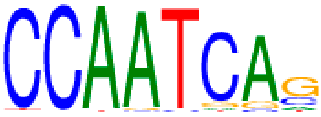	1.5e-07	NFYA
SEMA5A-AS1	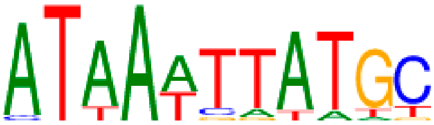	6.8e-08	POU6F1, T093398_1.02
CCDC138	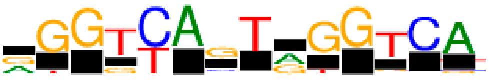	5.8e-06	NR1D1
NRIP1	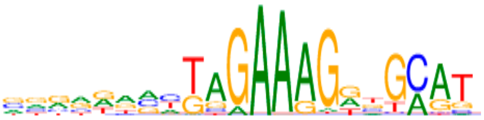 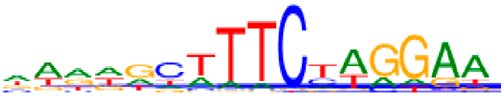	2.3e-08	ZNF184 + <br>BCL6 (transcriptional repressor which has emerged as a critical regulator of germinal centers (GC))
ARHGEF28	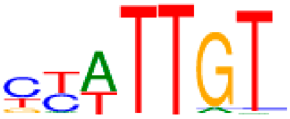	3.6e-09	SOX15
CLEC16A	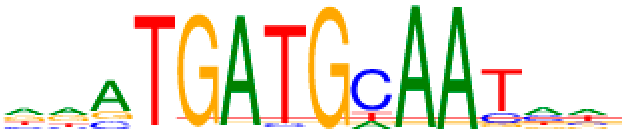	8.8e-09	ATF4

CDH17	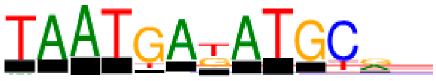	1.5e-06	POU6F1
FAM71D	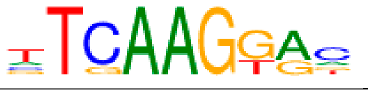	3.0e-07	NKX2-8
LINC02720	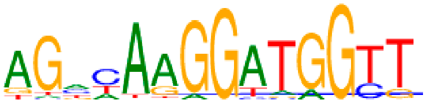	1.1e-09	ZNF324
PTGDR	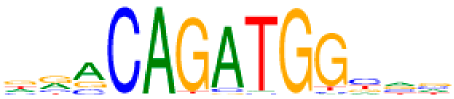	2.5e-10	NEUROD1
GPR15	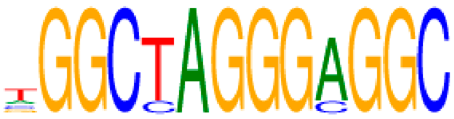	4.2e-12	Histone-lysine N-methyltransferase PRDM9
FABP6	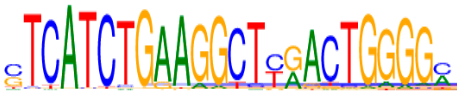	1.9e-06	ZNF304
MIR490	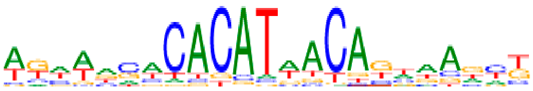	1.6e-08	EKLF, Erythroid krueppel-like transcription factor, HBFQTL6, INLU, KLF1_R328H_R2, Krueppel-like factor 1
LRRC52	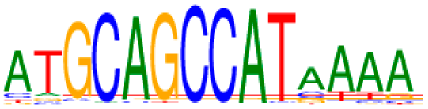	2.4e-11	ZNF280D
CHEK2P2	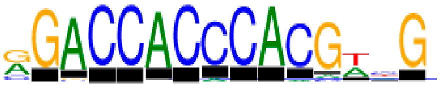	4.1e-11	GLI3
DSG2	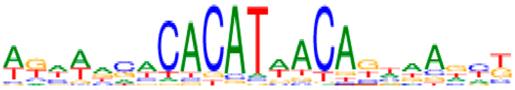	1.6e-08	EKLF, Erythroid krueppel-like transcription factor, HBFQTL6, INLU, KLF1_R328H_R2,

			Krueppel-like factor 1
KANK3	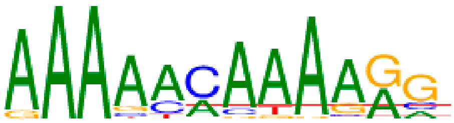	2.4e-10	ZNF182
LOC101927043	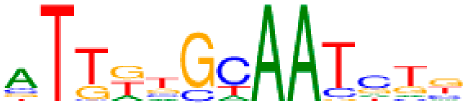	3.8e-09	CEBPG
C2orf61	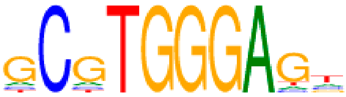	8.9e-08	Wilms tumor protein
PROKR2	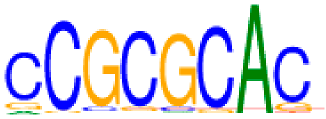	3.0e-10	ZBTB14
LINC01718	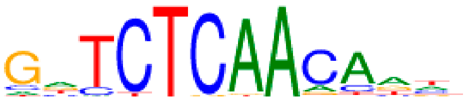	2.5e-10	ZNF627
CCDC134	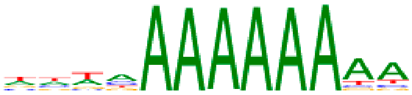<br>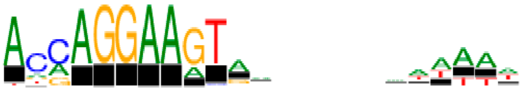	2.9e-13<br>8.8e-10	ZNF384<br>ELF3
IL1RAP	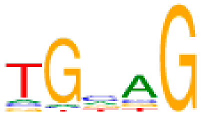<br>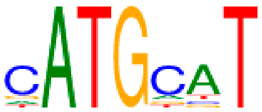	1.6e-06<br>1.5e-07	Zinc finger protein 217<br>Octamer-Binding Transcription Factor 1

FAT1	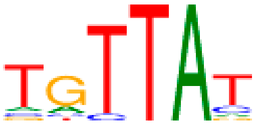	3.1e-08	Forkhead box protein O1
RHOH	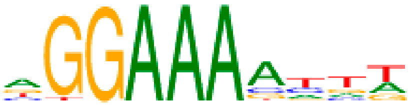	4.8e-08	NFATC4
MRPS30	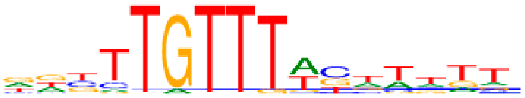<br>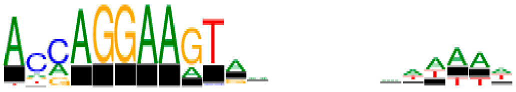<br>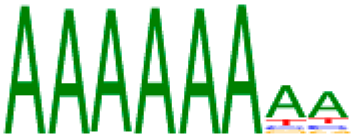	1.9e-10<br>5.0e-10<br>4.7e-08	FOXO1<br>ELF3<br>ZNF384
TTC33	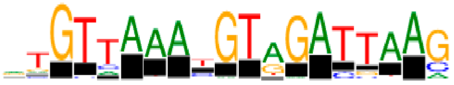	3.7e-09	ZNF232
FAXC	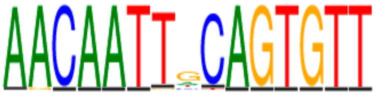	5.2e-08	SOX8

Supplementary Table S6. Common molecular targets for identified factors

Name of identified factors	Total number of common targets	Name of targeted molecular factors
DCP2, EIF4G3, PHF6, SF3B1	1	PLEKHA4
DCP2, GPR15, PHF6	1	miR-217-5p
DCP2, PHF6, SF3B1	2	CCNF, RAF1
DCP2, EIF4G3, SF3B1	1	YWHAG
EIF4G3, PHF6, SF3B1	7	MYCN, NAA40, NTRK1, PRKN, STAU1, TRIM67, ESR1
DCP2, GPR15	3	miR-148a-3p, miR-6740-3p, miR-20a-3p

GPR15, PHF6	8	miR-555, miR-4475, miR-143-3p, miR-2682-5p, miR-6758-5p, miR-6841-3p, miR-4780, miR-455-3p.
GPR15, SF3B1	1	miR-1243
EIF4G3, GPR15	4	miR-3924, miR-6868-3p, miR-4666a-5p, miR-1237-3p
DCP2, PHF6	9	SUFU, COIL, HNRNPA2B1, miR-3613-3p, miR-5094, miR-1283, miR-3117-5p, miR-4689, miR-377-3p
DCP2, SF3B1	10	PRPF40A, SRP9, SGF29, HDLBP, FANCD2, RBM8A, miR-330-3p, miR-5089-3p, miR-124-5p,
DCP2, EIF4G3	4	SNCA, DDX6, PSMC3, miR-4691-5p
PHF6, SF3B1	50	TRIM31, SNRNP200, RPL10, MAP1LC3B, MAGOH, ZRANB1, CAND1, UFL1, CUL3, BRD4, BRD7, H4C1, CHD4, NUPR1, LARP7, DDRGK1, RNF113A, PRPF8, EP300, RALY, NR2C2, H1-4, RPLP0, TP53BP1, ZBTB2, RECQL4, HSPA1A/HSPA1B, FBXW7, RPS8, KLF16, CHD3, HNRNPC, MYC, PHB1, CMTR1, RPL13, BIRC3, MEPCE, CSNK1A1, MECOM, RC3H1, RNF4, SMURF1, RC3H2, FZR1, STIP1, miR-452-5p, miR-193a-3p, miR-3611, miR-4477b
EIF4G3, PHF6	9	ESR2, CTR9, DYRK1A, RPL4, MRFAP1L1, HNRNPL, miR-6802-3p, miR-1278, miR-1301-5p
EIF4G3, SF3B1	16	VIRMA, YES1, EIF4A2, HSPA8, RPL19, GRWD1, SIRT7, FUS, EIF4E, BAP1, USP10, A1BG, LGR4, NCBP1, KIF23, miR-106a-3p
PHF6	1	SF3B1
