# Supplementary figures and images for: Exploring the Role of E6 and E7 Oncoproteins in Cervical Oncogenesis through MBD2/3-NuRD Complex Chromatin Remodeling
Source: Genes (Basel). 2024 Apr 27;15(5):560. doi: 10.3390/genes15050560 (PMC11121560; doi:10.3390/genes15050560)

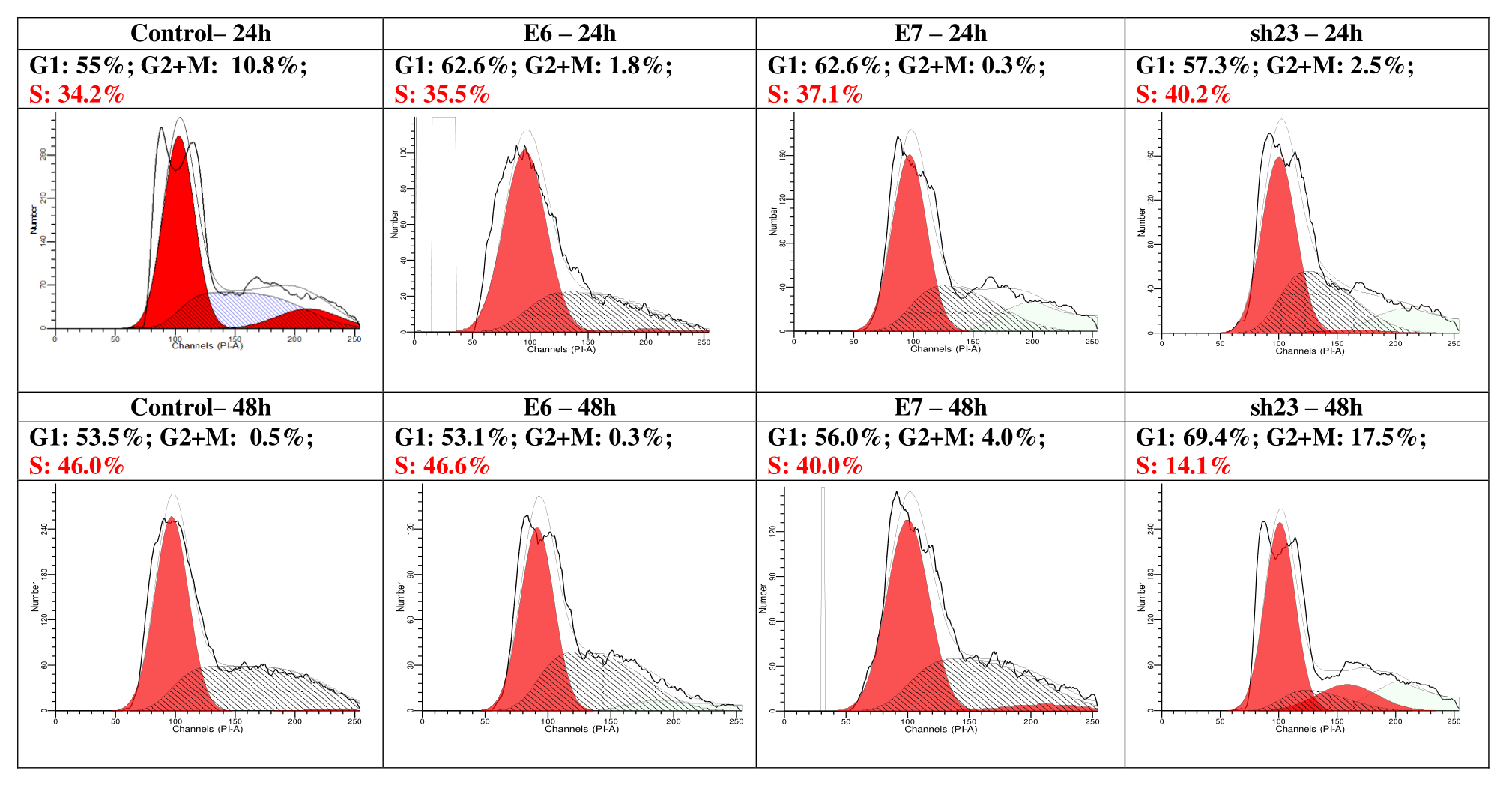

Supplement: Supplementary file 1 [file genes-15-00560-s001.zip › Supplementary Figure S1.tiff]

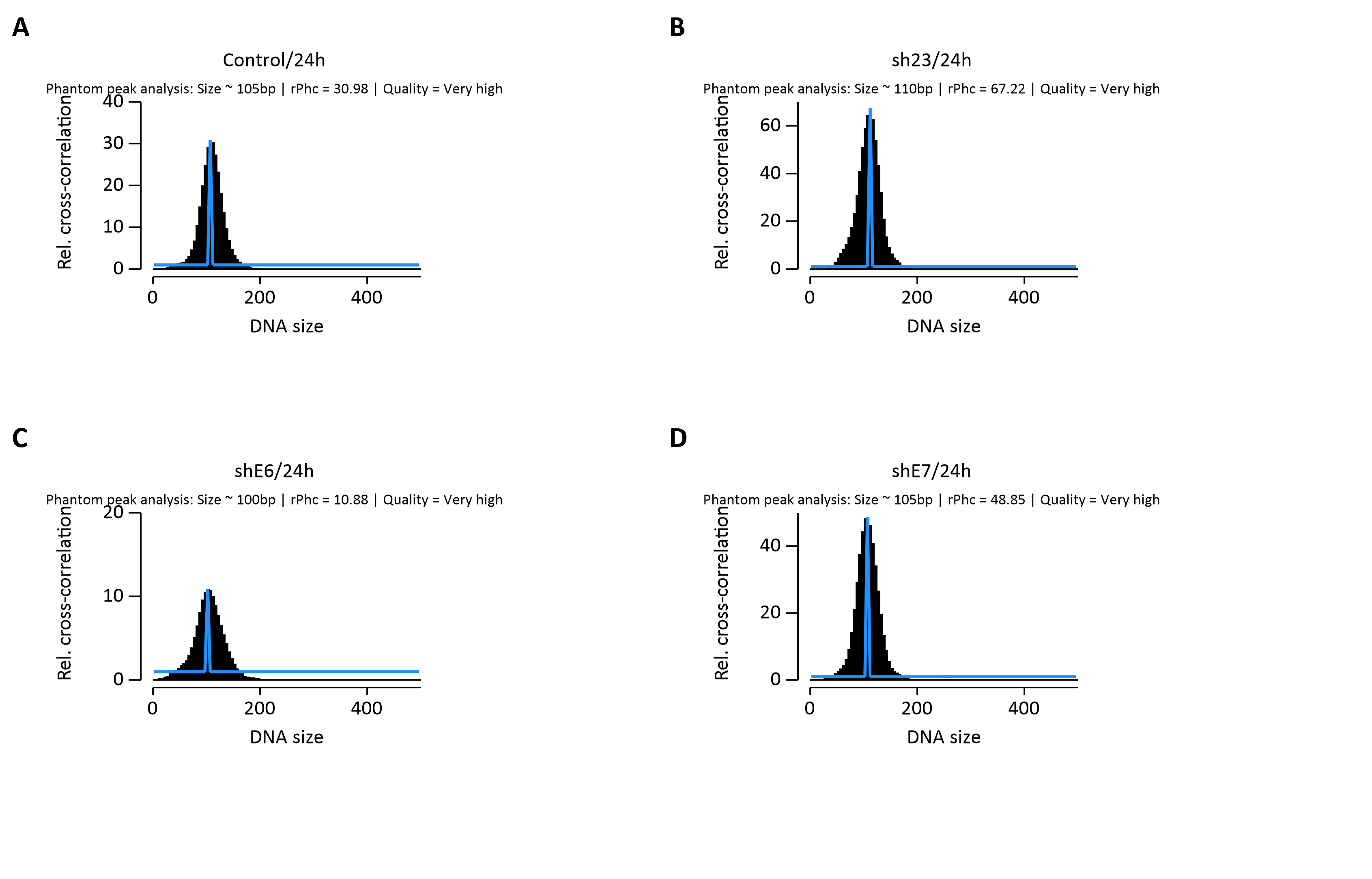

Supplement: Supplementary file 1 [file genes-15-00560-s001.zip › Supplementary Figure S2.tif]
